# A fully-automated, robust, and versatile algorithm for long-term budding yeast segmentation and tracking

**DOI:** 10.1371/journal.pone.0206395

**Published:** 2019-03-27

**Authors:** N. Ezgi Wood, Andreas Doncic

**Affiliations:** 1 Department of Cell Biology, UT Southwestern Medical Center, Dallas, Texas, United States of America; 2 Green Center for Systems Biology, UT Southwestern Medical Center, Dallas, Texas, United States of America; Pennsylvania State Hershey College of Medicine, UNITED STATES

## Abstract

Live cell time-lapse microscopy, a widely-used technique to study gene expression and protein dynamics in single cells, relies on segmentation and tracking of individual cells for data generation. The potential of the data that can be extracted from this technique is limited by the inability to accurately segment a large number of cells from such microscopy images and track them over long periods of time. Existing segmentation and tracking algorithms either require additional dyes or markers specific to segmentation or they are highly specific to one imaging condition and cell morphology and/or necessitate manual correction. Here we introduce a fully automated, fast and robust segmentation and tracking algorithm for budding yeast that overcomes these limitations. Full automatization is achieved through a novel automated seeding method, which first generates coarse seeds, then automatically fine-tunes cell boundaries using these seeds and automatically corrects segmentation mistakes. Our algorithm can accurately segment and track individual yeast cells without any specific dye or biomarker. Moreover, we show how existing channels devoted to a biological process of interest can be used to improve the segmentation. The algorithm is versatile in that it accurately segments not only cycling cells with smooth elliptical shapes, but also cells with arbitrary morphologies (e.g. sporulating and pheromone treated cells). In addition, the algorithm is independent of the specific imaging method (bright-field/phase) and objective used (40X/63X/100X). We validate our algorithm’s performance on 9 cases each entailing a different imaging condition, objective magnification and/or cell morphology. Taken together, our algorithm presents a powerful segmentation and tracking tool that can be adapted to numerous budding yeast single-cell studies.

## Introduction

Traditional life science methods that rely on the synchronization and homogenization of cell populations have been used with great success to address numerous questions; however, they mask dynamic cellular events such as oscillations, all-or-none switches, and bistable states [1–5]. To capture and study such behaviors, the process of interest should be followed over time at single cell resolution [6–8]. A widely used method to achieve this spatial and temporal resolution is live-cell time-lapse microscopy [9], which has two general requirements for extracting single-cell data: First, single-cell boundaries have to be identified for each time-point (segmentation), and second, cells have to be tracked over time across the frames (tracking) [10, 11].

One of the widely-used model organisms in live-cell microscopy is budding yeast *Sacchromyces cerevisiae*, which is easy to handle, has tractable genetics, and a short generation time [12, 13]. Most importantly in the context of image analysis, budding yeast cells have smooth cell boundaries and are mostly stationary while growing, which can be exploited by segmentation and tracking algorithms. Thus, in contrast to many mammalian segmentation approaches that segment only the nucleus, use dyes to stain the cytoplasm [14–17], use manual cell tracking [18] or extract features using segmentation-free approaches [19], we expect yeast segmentation to be completely accurate using only phase or bright-field images. Hence, budding yeast segmentation and tracking pose a complex optimization problem in which we strive to simultaneously achieve automation, accuracy, and general applicability with no or limited use of biomarkers.

Several different methods and algorithms have been created to segment and track yeast cells. To reach high accuracy, some of these algorithms rely on images where cell boundaries and/or the cell nuclei are stained [20–22]. However, with staining, one or several fluorescent channels are ‘occupied’, which limits the number of available channels that could be used to collect information about cellular processes [23]. In addition, using fluorescent light for segmentation increases the risk for photo-toxicity and bleaching [24]. Thus, it is desirable to segment and track cells using only bright-field or phase images.

Another commonly used method, ‘2D active contours’, fits parametrized curves to cell boundaries [25]. Existing yeast segmentation algorithms using this method typically take advantage of the elliptical shape of cycling yeast cells [26–28]. Another way to take advantage of the prior information on cell shape is to create a shape library where shapes from an ellipse library and cells are matched [29]. Although these methods can be very accurate, they tend to be computationally expensive [29], and, to the best of our knowledge, they are not tested on any non-ellipsoidal morphologies, e.g. sporulating or pheromone treated cells. Moreover, in many cases they have to be fine-tuned to the specific experimental setup used [27, 29].

Here we present a fully automated segmentation and tracking algorithm for budding yeast cells. The algorithm builds on our previously published algorithm [30], significantly improves its accuracy and speed, and fully automatizes it by introducing a novel automated seeding step. This seeding step incorporates a new way for automated cell boundary fine-tuning and automated correction of segmentation errors. Our algorithm is parallelizable, and thus fast, and segments arbitrary cell shapes with high accuracy. Our algorithm does not rely on segmentation specific staining or markers. Still, we show how information about cell locations can be incorporated into the segmentation algorithm using fluorescent channels that are *not* devoted to segmentation. To demonstrate the versatility of our algorithm we validate it on 9 different example cases each with a different cell morphology, objective magnification and/or imaging method (phase / bright-field). In addition, we compare its performance to other algorithms by using a publicly available benchmark.

## Results

### Automated seeding

When segmenting yeast cells over time, it is advantageous to start at the last time-point and segment the images backwards in time [30], because all cells are present at the last time point due to the immobility of yeast cells. Thus, instead of attempting the harder problem of detecting newborn cells (buds), we only have to follow existing cells backwards in time until they are born (disappear). To segment the cells, we therefore need an initial segmentation of the last time-point, which is fed to the main algorithm that uses the segmentation of the previous time point as the seed for the next time point.

This seeding step was previously a bottleneck since it was semi-automated and required user-input. To fully automate the segmentation algorithm, we developed a novel method to automate this seeding step. Here we present the general outline of this method. For a detailed explanation see S1 Text and the accompanying annotated software (S1 Codes and Example Images).

**Fig 1:**
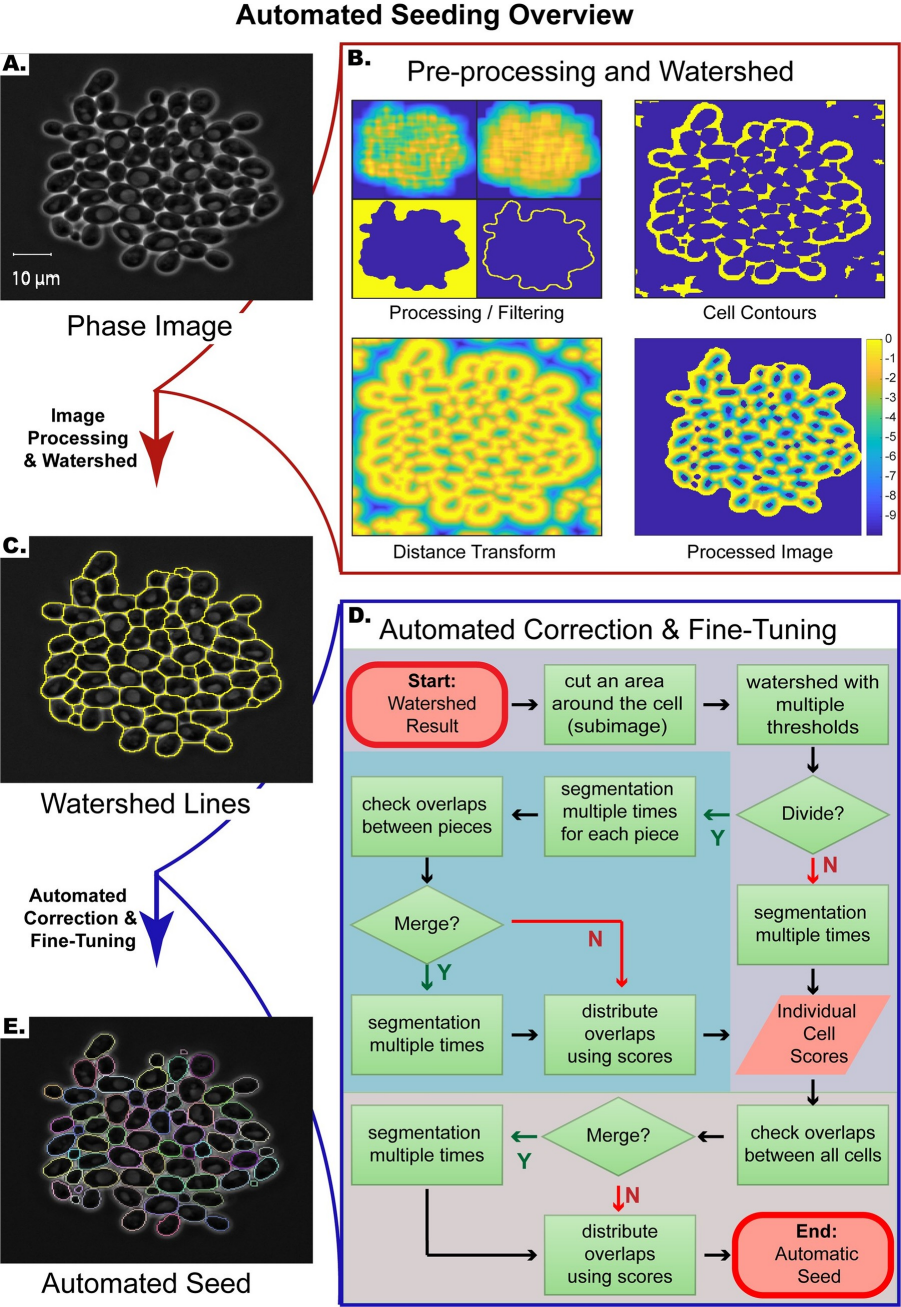
Automated seeding overview. **(A)** Example phase image. **(B)** First step of automated seeding algorithm: Pre-processing and watershed. In this step, the watershed transform is applied to the processed image. **(C)** Phase image with watershed lines (yellow). **(D)** Flowchart of the second step of automated seeding: Automated correction and fine-tuning. At this step, the cell boundaries are automatically fine-tuned, and segmentation errors are automatically corrected. **(E)** The result of the automated seeding step. Each cell boundary is marked with a different color.

The automated seeding algorithm has two main steps (Fig 1): First, watershed algorithm is applied to the pre-processed image of the last time point (Fig 1A–1C). Second, the resulting watershed lines are automatically fine-tuned, and segmentation mistakes are automatically corrected (Fig 1D and 1E).

#### Pre-processing and watershed

**Fig 2:**
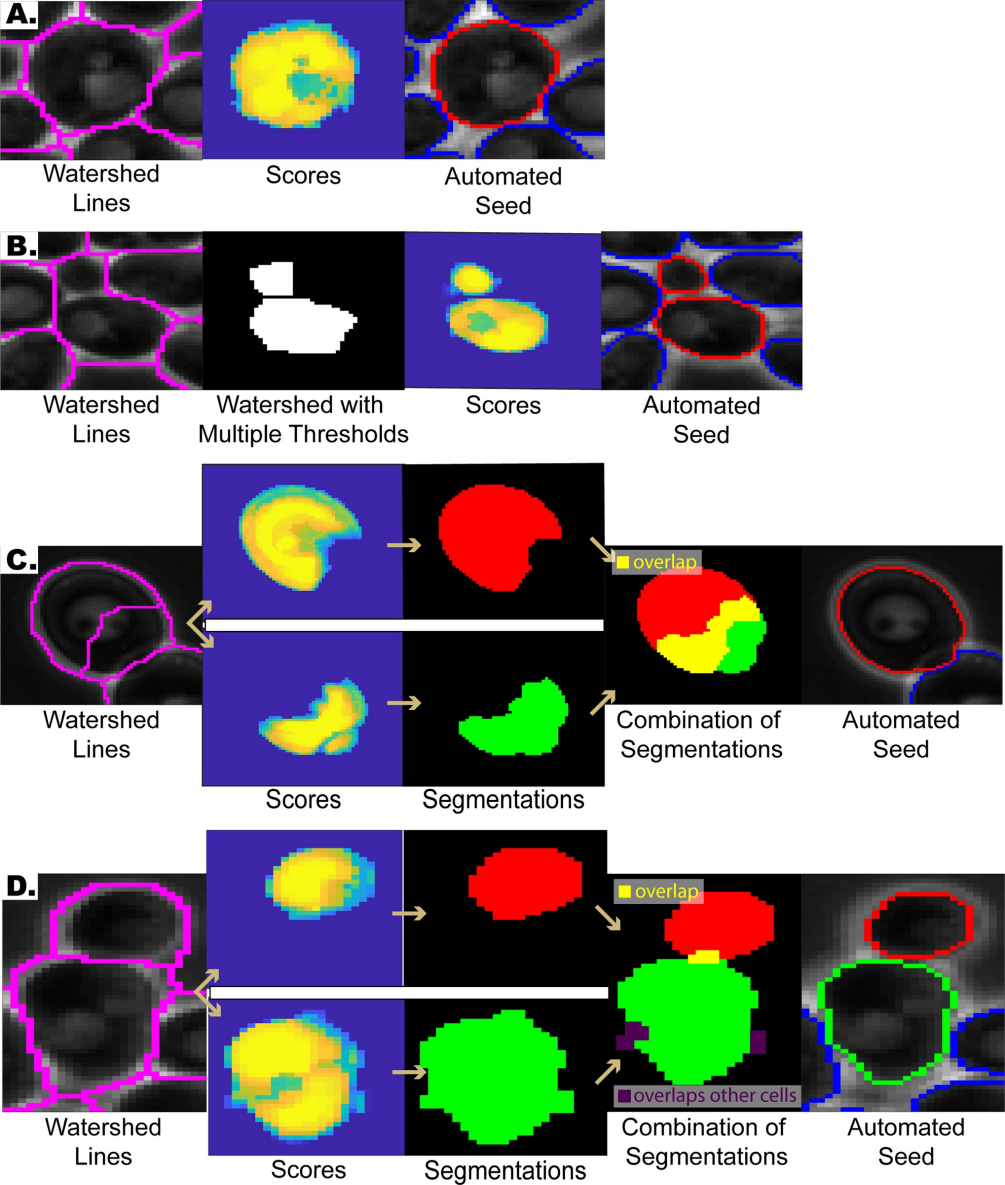
Automated correction & fine-tuning step examples. **(A)** Refining cell boundaries: The watershed lines do not mark the exact cell boundaries (first column, magenta). Our algorithm automatically fine-tunes these watershed lines and marks the correct cell boundary (third column, red). **(B)** Under-segmentation correction: Sometimes the watershed lines merge multiple cells (first column, magenta). Such mistakes are detected and corrected automatically (fourth column, red). **(C)** Over-segmentation correction: Sometimes the watershed lines divide a cell into multiple pieces (first column). After applying the segmentation subroutine several times, each piece converges towards the correct cell segmentation and thus the pieces overlap significantly (fourth column). If the overlap between two pieces are above a certain threshold, then they are merged (fifth column, red). **(D)** Distribution of Overlaps: The algorithm sometimes assigns the same pixels to the segmentations of adjacent cells (Also see section *Distribution of overlapping initial segmentations*), which leads to overlapping cell segmentations. Such overlaps (fourth column, yellow) are distributed among the cells based on their scores.

During this step, the image is processed before the application of the watershed transform, with the aim of getting only one local minimum at each cell interior, so that each cell area will be associated with one segmented region after the application of the watershed transform. To this end, the image is first coarsely segmented to determine the cell and non-cell (background) regions of the image (Fig 1B, Processing/Filtering, binary image on the bottom left). Based on this coarse segmentation, the algorithm only focuses on the cell colonies. Next, cell contours and interstices are identified by exploiting the fact that they are brighter than the background pixels and cell interiors (Fig 1B, Cell Contours). To detect such pixels, we use mean and standard deviation filtering (Fig 1B, Processing/Filtering, top images) and label pixels that are brighter than their surroundings as cell contour pixels. Once these cell contour pixels are determined, we apply a distance transform to this binary image and further process the transformed image (Fig 1B, Distance Transform and Processed Image). Next, we apply a watershed transform to the resulting image (Fig 1C). Note that even though the watershed lines will separate the cells, they do not mark the exact boundaries (Fig 2A). In addition, sometimes multiple, or lack of, local minima within cells leads to situations where multiple cells are merged as one or a cell is divided into multiple regions (under/over-segmentation, Fig 2B and 2C).

#### Automated correction and fine-tuning

To refine the cell boundaries and to automatically correct segmentation mistakes, we implemented the second step (Fig 1D), which takes as the input the watershed result from the previous step (Fig 1C) and gives as the output the final automated seed (Fig 1E). For each cell, this algorithm focuses on a subimage containing the putative cell region determined by the watershed lines. First, the algorithm checks whether the putative cell area contains more than one cell (under-segmentation), i.e. whether the putative cell region needs to be divided. This is achieved by testing the stability of the putative cell location under different parameters: the previous pre-processing and watershed step is applied on the subimage, but this time with multiple thresholds for determining the cell contour pixels. Each threshold has a ‘vote’ for assigning a pixel as a cell pixel or a non-cell pixel, which eventually determines whether the area will be divided. If the putative cell is divided, then each piece is treated separately as an independent cell (Fig 1D, blue box). Next, the subimage is segmented using a version of the previously published segmentation subroutine [30] (See S1 Text section *Review of the previously published subroutine*.), in which the image is segmented multiple rounds using the result of the previous segmentation as the seed for the next segmentation. Through these segmentation iterations, the coarse seed obtained by the watershed transform converges onto the correct cell boundaries, thereby fine-tuning the segmentation. Also, this step generates a *score* for each putative cell, which is an image carrying weights representing how likely each pixel belongs to the cell. These scores are used in case the same pixels are assigned to adjacent cells, leading to overlapping cell segmentations. If these overlaps are small, the algorithm distributes them among the cells based on the scores generated at the segmentation step (Fig 2D. See also section *Distribution of overlapping initial segmentations*.). If the intersection between two putative cell segmentations is above a certain threshold, then the algorithm merges these two regions to correct over-segmentation mistakes (Fig 2C).

To test our automated seeding step, we applied it to a wide range of example cases: (1) cycling cells imaged by phase contrast with 40X objective and (2) 63X objective, (3) sporulating cells imaged by phase contrast with 40X objective, (4) *cln1 cln2 cln3* cells imaged by phase contrast with 63X objective, (5–8) *cln1 cln2 cln3* cells exposed to 3, 6, 9 and 12nM mating pheromone (α-factor) imaged by phase contrast with 63X objective, and (9) bright-field images of cycling cells imaged with 40X objective. Note that bright-field images were briefly processed before feeding them into the seeding algorithm (see S1 Text).

Next, the segmentations were scored manually (Table 1). Cells whose area were correctly segmented over 95% were scored as ‘correct’. A significant fraction of the segmentation mistakes was minor, and they were automatically corrected within 10 time points after the seed was fed into the segmentation and tracking algorithm (Table 1. See also section *Robustness of Segmentation*.). Note that most of the seeding errors emerged from cells with ambiguous cell boundaries, such as dead cells.

**Table 1:** Automated seeding performance.

	Initial fraction of correctly segmented cells %	Final fraction of correctly segmented cells after 10 time points	Average # of time points needed for correction	# cells	# Fields of View
**40X –cycling**	95.9%	98.6%	3.6	435	2
**63X –cycling**	95.2%	97.3%	5.8	293	3
**40X- sporulating**	96.6%	96.9%	2	352	2
**63X – 0 nM**	92.5%	-	-	67	3
**63X– 3nM**	93.7%	95.8%	5.3	143	4
**63X – 6 nM**	90.2%	-	-	102	4
**63X – 9 nM**	86.24%	89.9%	5.2	109	6
**63X –12nM**	71.64%	-	-	67	5
**Bright-field**	95.5%	-	-	308	2

0, 3, 6, 9, 12 nM refer to α-factor concentrations used for treating *cln1cln2cln3* cells. #:Number

-: no cells are corrected

Finally, we implemented a correction step after the automatic seeding, where faulty seeds can be adjusted or removed semi-automatically. For screening or large-scale applications this step can be omitted with little loss of accuracy.

### Computational performance

When segmenting an image, the algorithm first segments each cell independent of other cells by focusing on a subimage containing a neighborhood around the cell’s seed. Through parallelization of this step, we significantly improved the speed of our algorithm.

**Fig 3:**
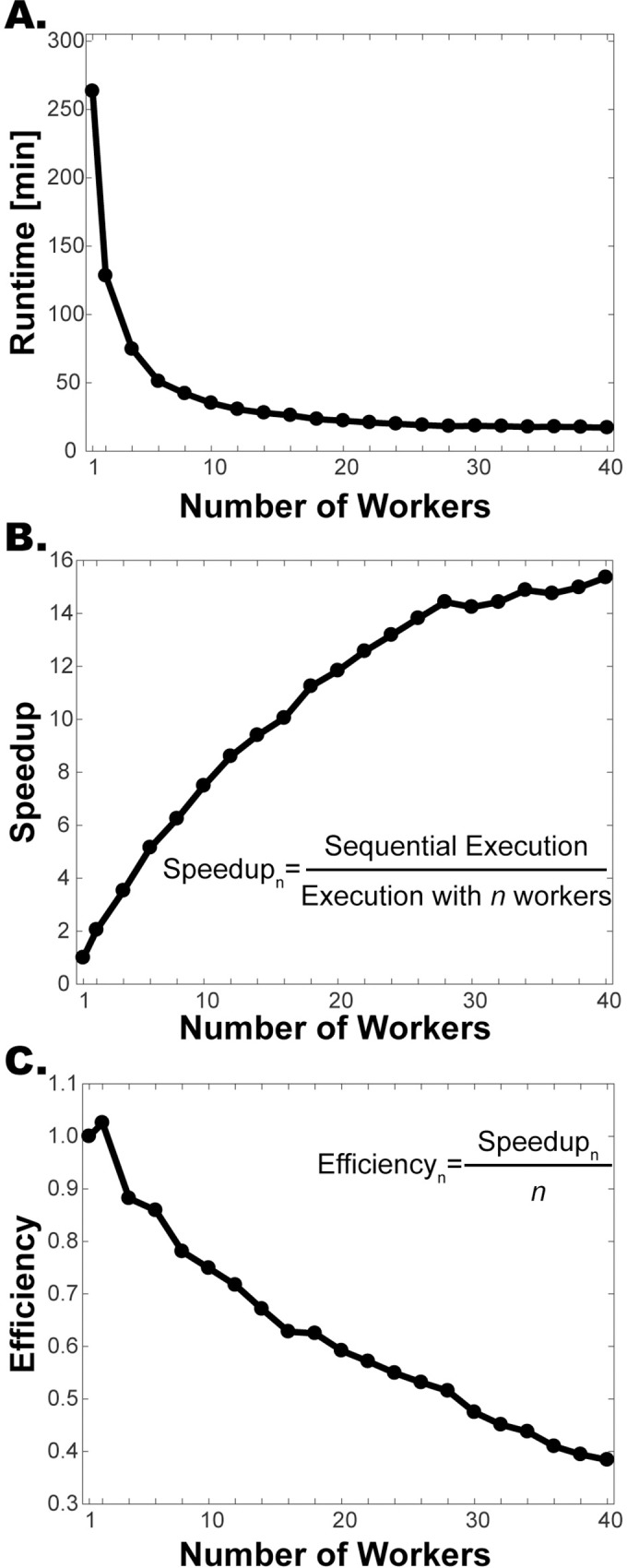
Time gain, speedup and efficiency achieved by parallelization. An example field of view imaged over 10 hours (200 time points, 360 cells at the last time point) was segmented sequentially and in parallel with varying number of workers. **(A)** Runtimes. **(B)** Speedup is calculated by dividing the sequential execution time by the parallel execution time. With 40 workers the algorithm runs 15.4 times faster. **(C)** Efficiency is the speedup per processor. Note that the efficiency goes down as the number of processors increases.

To demonstrate the gain in runtime we segmented an example time-series of images sequentially without parallelization and in parallel with varying number of workers (i.e. parallel processors). The example time-series had 200 images and 360 cells on the last image, which amounted to 25377 segmentation events. With 40 workers the algorithm runs about15-times faster (263 min vs 17 min, Fig 3A). Note that after about 26 workers, there is no significant difference in runtime, since the time gain is limited by the longest serial job. Also, overhead communication time increases with increasing number of workers offsetting the time gain.

We also calculated the performance measures *speedup* and *efficiency* [31]. The *speedup* is the ratio of the runtime without parallelization to runtime with *n* processors. The speedup increases as the number of workers increases, but eventually levels off (Fig 3B). Next, we calculated the *efficiency*, which is the speedup divided by the number of processors. This gives a measure of how much each processor is used on average [31]. The efficiency is highest for 2 processors and it decreases as the number of processors are increased (Fig 3C).

Personal computers with quad processing cores can run successfully with four workers, which sped up the runtime about 3.5 times with the example images. Thus, even in the absence of a computer cluster, one can significantly improve the efficiency of the algorithm on a personal computer.

### Distribution of overlapping initial segmentations

**Fig 4:**
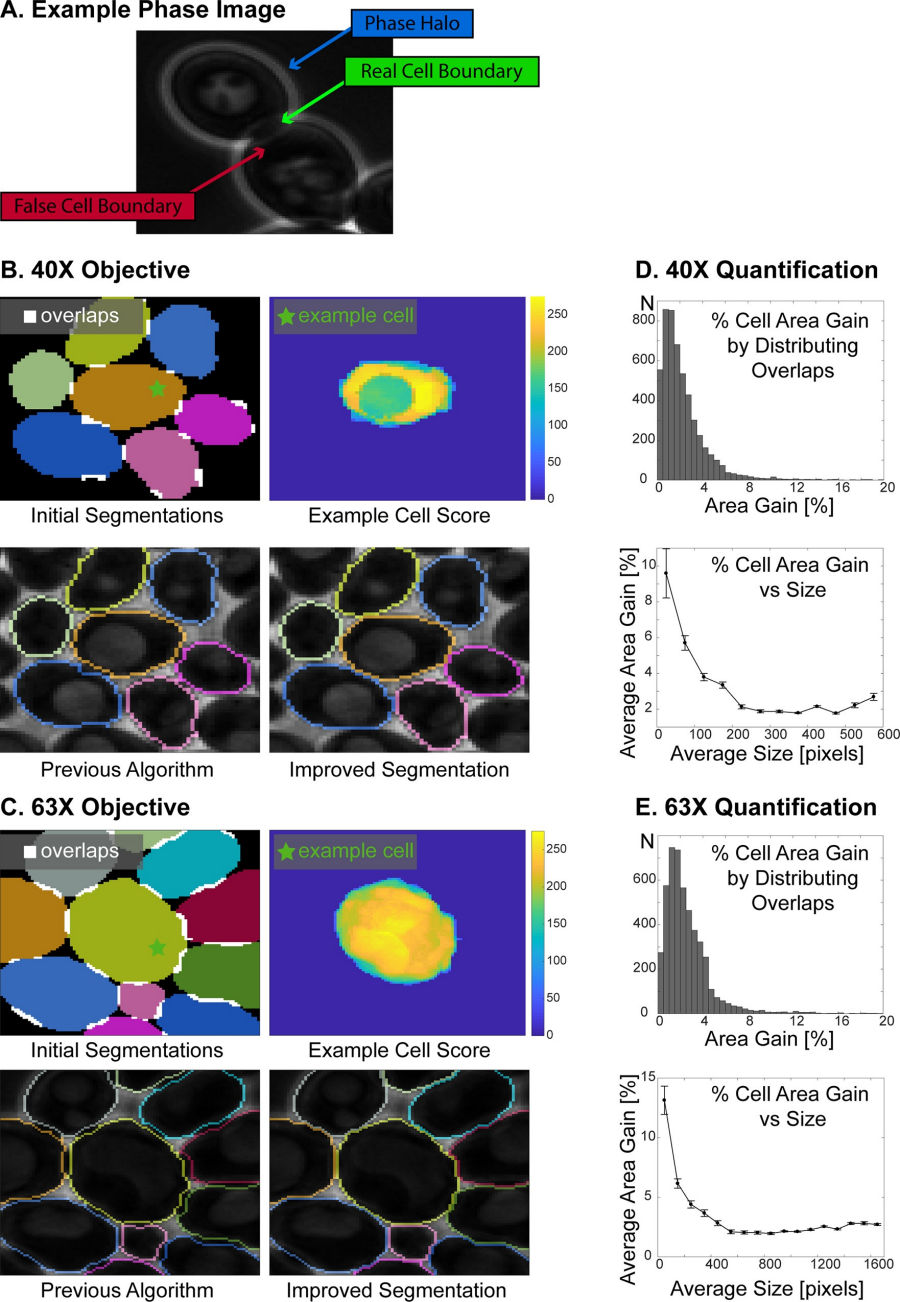
Distribution of overlapping initial segmentations. **(A**) Example phase image showing two neighboring cells: There is a bright halo (phase halo) around the cells in phase images. When cells are touching, these halos can create a false cell boundary detected by the algorithm. Thus, the algorithm sometimes assigns the same pixels to neighboring cells leading to overlapping cell segmentations. **(B-C)** Example cells imaged with 40X **(B)** and 63X **(C)** objectives. Initial Segmentations: Overlaps between the initial segmentations of the neighboring cells are highlighted as white areas. Each cell segmentation is represented with a different color. Example Cell Score: Each individual cell has a cell score, which carries weights for whether a pixel should belong to the cell. Previous Algorithm: Overlapping regions among the initial segmentations were excluded from the segmentation in the previous algorithm [30]. Improved Segmentation: In the new algorithm such overlapping regions are distributed among the cells based on their scores, which significantly improves the segmentation at the cell boundaries. **(D-E)** Comparison of cell areas with and without distributing the overlapping regions for 40X **(D)** and 63X **(E)** objectives. Cells imaged over 10 hours (100 time points) were segmented with and without distributing the overlapping segmented regions. By distributing these intersections, the majority of cells gained cell area (75% for 40X and 97% for 63X. See Table 2.). Percent area gain is calculated by dividing the difference of the cell area with and without distributing the intersections by the area with distributing the intersections and then multiplying the result by 100. Next, the average percent cell area gain versus average size is plotted. To this end, cell sizes are grouped in 50-pixel increments (40X) or in 100-pixel increments (63X). The average size of each group is plotted against the average percent size gain in that group. The error bars show the standard error of the mean. Note that for small cells (buds) area gain percentage is higher than mother cells.

Phase contrast microscopy, which produces a sharp contrast between cells and background, is in general preferable for yeast segmentation and tracking. Yet phase imaging always produces a phase halo around objects [32] that might produce ‘false’ cell boundaries in the context of densely packed cells (Fig 4A). When these ‘false’ boundaries invade the neighboring cells, the segmentation algorithm might assign the same pixels to multiple cells in a way that their segmentations overlap (Fig 4B and 4C, white pixels in Initial Segmentations), even though the cells are not physically overlapping.

**Table 2:** Area gain by distribution of overlapping pixels.

	% of Segmentations with Area Gain*	% Cell Area Gain (given there is a gain)		Pixel Gain (given there is a gain)		# Data Points with Area Gain	# Fields of View
		Mean	Std	Mean	Std		
**40X cycling**	74.6%	2.3%	2.6%	6.8	5.4	5154	2
**63X cycling**	96.7%	2.7%	2.8%	22.4	16.7	4838	3
**63X** **0 nM**	76.7%	2.0%	2.7%	18.3	17.1	7557	3
**63X** **3 nM**	84.8%	2.3%	3.0%	23.2	24.6	10899	2
**63X** **6 nM**	93.3%	1.9%	2.5%	21.0	20.8	7975	3
**63X** **9 nM**	82.2%	1.4%	1.8%	13.3	15.0	8324	3
**63X** **12 nM**	82.1%	1.5%	1.6%	12.0	9.8	5531	3

0, 3, 6, 9, 12 nM refer to α-factor concentrations used for treating *cln1cln2cln3* cells. #:Number

*For the percent area gain calculations presented in Table 2 only correctly segmented cells are used.

In the initial version of our algorithm [30], such overlapping segmented regions were excluded from the segmentation (Fig 4B and 4C, Previous Algorithm). To improve the segmentation accuracy, we developed a method to segment these overlapping segmented areas as well (Fig 4B and 4C, Improved Segmentation). After the cells are segmented individually to get the initial segmentations, the cell segmentations are compared to detect the overlapping pixels. Next, any such overlapping pixels are distributed based on the scores among cells with overlapping segmentations. Note that this step is also implemented for automatic seeding (Figs 1D and 2C).

To validate this procedure, we segmented cycling cells imaged for 10 hours (100 time points) with 40X and 63X objectives with distributing the overlapping initial segmentations or without distributing but discarding them. Distributing the overlapping segmented regions significantly improved the segmentation as measured by the increase of correctly segmented cell area (Fig 4B–4E, S1 and S2 Movies). Specifically, the vast majority of cells had a non-zero area gain (75%/97% for 40X/63X, Table 2). The cells with an area gain, had increased their area 2.3 ± 2.6% (40X, N_40X_ = 5154) and 2.7 ± 2.8% (63X, N_63X_ = 4838) on average. The percent cell area gain is calculated as:

$$\%\;cell\;area\;gain=\frac{{Area}_{with\;distributing\;the\;overlaps}-{Area}_{without\;distributing\;the\;overlaps}}{{Area}_{with\;distributing\;the\;overlaps}}\times 100$$

**Fig 5:**
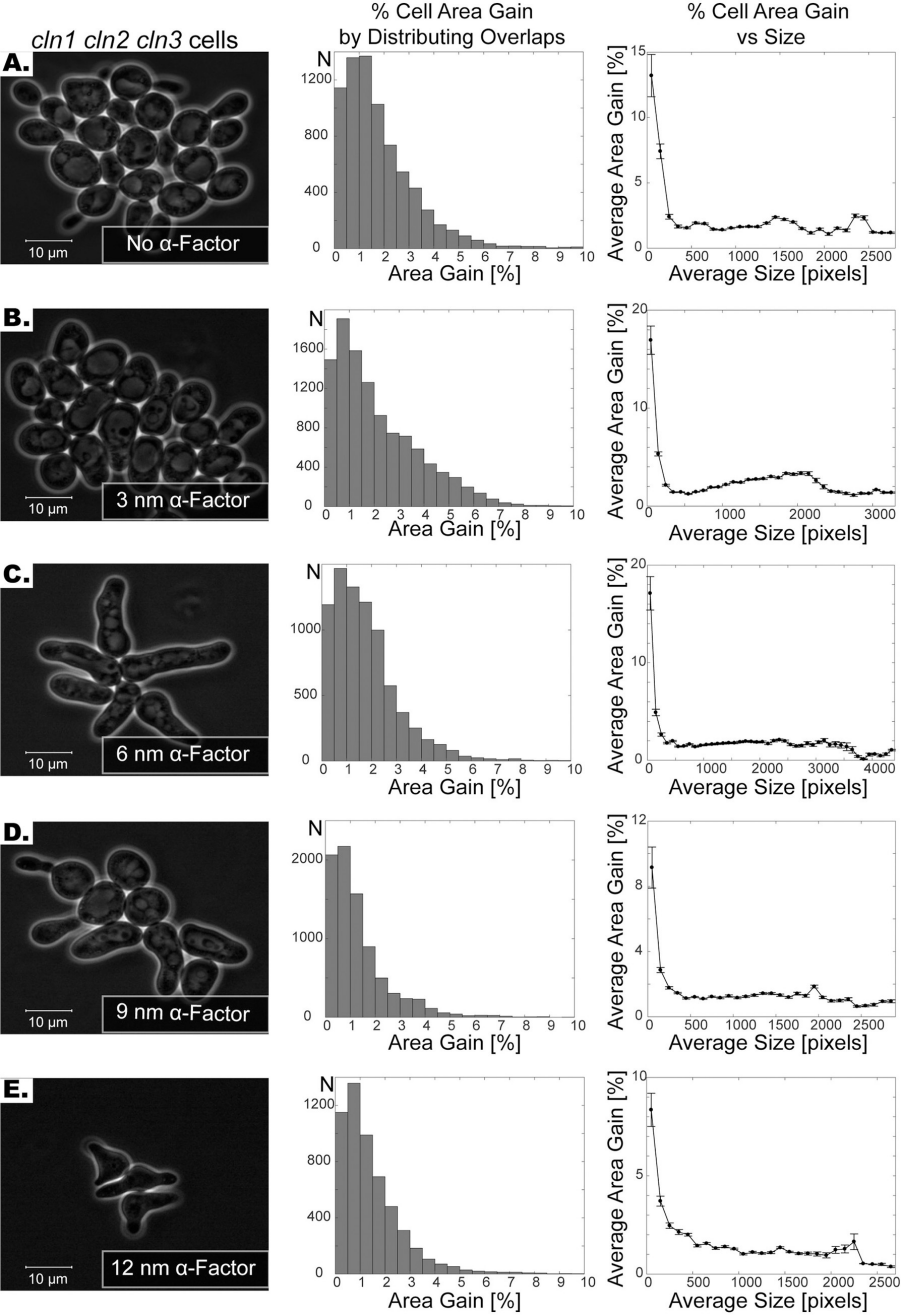
Segmentation of cells subject to varying levels of pheromone treatment. **(A-E)** First column shows the phase images of *cln1 cln2 cln3* cells without α-factor **(A)** and with varying levels of α-factor treatment **(B-E)**. Note that the shapes get progressively more irregular as the concentration of the α-factor increases. Second column shows the histogram of percent area gain by distributing the overlapping segmentation regions. Note that histograms are capped at 10%. Third column shows the relationship between size of the cell and the percent cell area gain. The cell sizes are grouped in 100-pixel increments. The average size of each group is plotted against the average percent size gain in that group. The error bars show the standard error of the mean. Note that for small cells area gain percentage is higher than that for larger cells.

We also tested this correction method for cells with abnormal morphologies. To this end we used a yeast strain that lacks two out of three G1 cyclins (*cln1cln3*) and where the third (*cln2*) was conditionally expressed in our microfluidics-based imaging platform. Specifically, we grew cells for one hour before we arrested the cell cycle and added variable amounts of mating pheromone (0, 3, 6, 9, or 12 nM α-factor) which lead to various yeast morphologies (Fig 5A–5E, S3–S7 Movies) [33, 34]. By distributing the overlapping initial segmentations, here we noticed again a significant area gain (Table 2). Taken together, this demonstrates that the boundary correction method works and is robust across varying conditions.

Note that the distribution of overlapping initial segmentations has a negligible computational cost: With the addition of steps required for distribution of overlaps the algorithm took only 1.7 minutes longer on the example field of view used in the Section *Computational Performance* with 4 workers (79.0 min vs 80.77 min).

**Fig 6:**
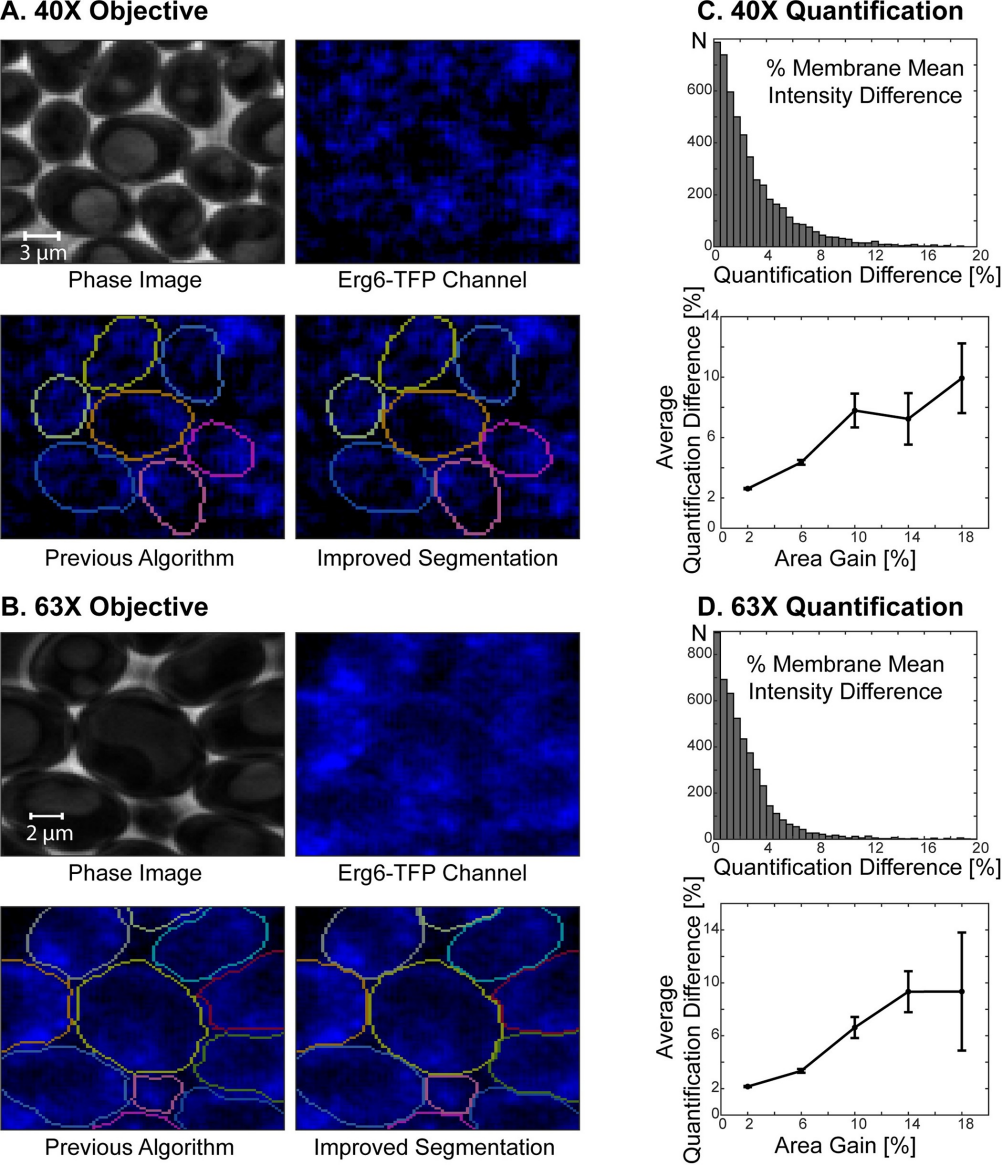
Quantification of the Erg6-TFP intensity at the cell periphery. **(A-B)** Example cells imaged with 40X **(A)** and 63X **(B)** objectives. The cell segmentations with the previous algorithm (without distributing the overlapping initial segmentations, but by removing them) and with the new algorithm (with distributing the overlapping initial segmentations) are shown side-by-side. Note that the cells are the same cells as shown in Fig 4. **(C-D)** Comparison of the Erg6-TFP mean intensity at the cell periphery with and without distribution the overlaps for 40X **(C)** and 63X **(D)** objectives. The same cells as in Fig 4 are used for this quantification. Percent quantification difference is calculated by dividing the absolute value of the quantification difference by the quantification with distributing the overlaps and then multiplying by 100. Next, the average percent cell area gain versus the average percent quantification difference is plotted. To this end the cells are grouped in 4% cell area gain increments and the average percent quantification difference is plotted against the mean of each group. The error bars show the standard error of the mean.

Although the percent cell area gain is 1.4–2.3% when averaged over all cells, the percent area gain can go up to 10–20% when the gains of smaller cells are averaged (Figs 4 and 5). More importantly, the distribution of overlapping segmentations significantly improves the segmentation of cells at the cell boundaries, thus enabling cell periphery localization quantification, which would be unreliable without distributing the overlapping initial segmentations. To show that the quantification of biomarker intensity significantly changes with distribution of the overlaps, we quantified the mean intensity of the Erg6-TFP at the cell periphery. Erg6 is an enzyme required for ergosterol synthesis and localizes primarily to lipid droplets [35, 36]. For the quantification, we used the same 40X and 63X cells reported in Table 2 and Fig 4. In particular, we calculated the mean intensity of the processed TFP-channel image on the 2-pixel thick cell periphery both with and without distributing the overlapping initial segmentations (See S1 Text for details.). Next, the percent quantification difference is calculated by

$$\%\;Quant.Difference=\frac{abs({Quant.}_{with\;distributing\;the\;overlaps}-{Quant.}_{without\;distributing\;the\;overlaps})}{{Quant.}_{with\;distributing\;the\;overlaps}}\times 100,$$

where *Quant*. stands for quantification and *abs* for absolute value. We show that the distribution of overlaps leads to a significant difference of the quantification of the biomarker intensity at the cell periphery, especially for cells with a higher area gain (Table 3, Fig 6). More specifically, 99.2% (40X, N = 5154) and 97.7% (63X, N = 4843) of the cells had a quantification difference of the Erg6-TFP signal at the cell periphery (Table 3). The percent quantification difference is about 3% when averaged over all cells, however, it goes up to 10% when averaged over cells with higher area gain (Fig 6). Thus, distribution of overlaps improves the data extracted from fluorescent channels and enables accurate cell periphery localization analysis.

**Table 3:** Quantification difference in mean membrane intensity.

	% of Quantifications with a Difference (for cells with an area gain)	% Quantification Difference (given there is a difference)		# Data Points with Quantification Difference	# Fields of View
		Mean	Std		
**40X cycling**	99.2	3.0%	3.4%	5112	2
**63X cycling**	97.7	2.7%	6.4%	4726	3

### Robustness of segmentation

The ability of a segmentation algorithm to correct an error is a key requirement for correct segmentation over a large number of time points. Otherwise, once an error is made, for example due to an unexpectedly large movement of a cell or a bad focus at one time point, it will linger throughout the segmentation of consecutive time points and errors will accumulate. Our algorithm can correct such errors, since it is robust to perturbations in the seed, i.e. even if there is a segmentation error at one time point, when the algorithm is segmenting the next time point using the previous wrong segmentation as a seed, it can still recover the correct cell boundaries.

**Fig 7:**
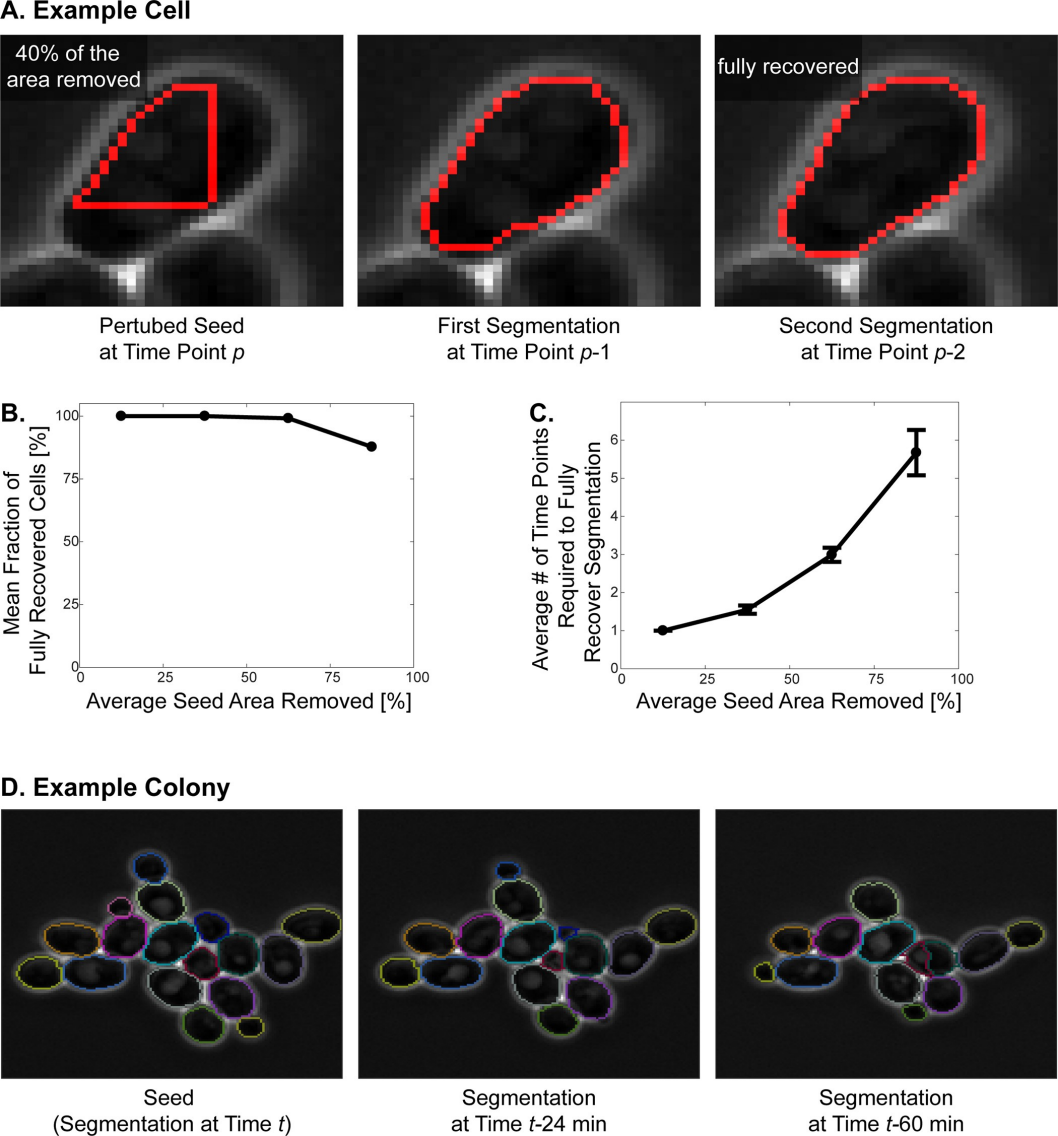
Robustness of the segmentation algorithm. **Robustness to errors in the seed. (A)** Example cell: The seed of the example cell is perturbed by randomly removing 40% of the seed. The algorithm uses this perturbed seed to segment the cell at time point *p*-1 and recovers the cell with only minor mistakes. The algorithm fully recovers the cell in two time points. Note that the algorithm segments the cells backwards in time, thus time points (i.e. frame numbers) are decreasing. **(B)** The seeds of 340 cells were perturbed by randomly removing 10–90% of the seed. The cells are grouped based on the severity of perturbation, i.e. percent seed area removed, in 25% increments. Mean fraction of fully recovered cells is plotted for each group. Note that out of 340 cells, only 9 of them were not recovered by the algorithm. **(C)** The cells are grouped based on the perturbation in 25% increments and the average number of time points required to fully recover the correct cell segmentation is plotted for each group. Number of time points required to fully recover the cells increase with the severity of the seed perturbation. The error bars show standard error of the mean. Robustness to time interval between frames. **(D)** Example colony used for the quantification presented in Table 4. The correct segmentation at time *t* is used as a seed to segment the images taken at *t-24* min and *t-60* min. All cells are segmented accurately when the time interval between the seed and the image is 24 minutes. However, when this interval is raised to 60 minutes, a major error is introduced (See the over-segmented cell in red and green.).

To test the robustness of our algorithm to errors in the seed (i.e. segmentation of the previous time point), we randomly picked 340 actively cycling cells imaged every 3 minutes with 40X objective. Next, we perturbed their seed (i.e. segmentation of the last time point) by removing 10–90% of the total cell area (Fig 7A). Then, we ran the segmentation algorithm with these perturbed seeds.

**Table 4:** Segmentation accuracy with respect to the time interval between frames.

Time interval [min]	Total # of cells	Fraction of accurate segmentations	Fraction of minor segmentation errors	Fraction of major segmentation errors	# fields of view
**3**	140	100%	0%	0%	2
**12**	137	100%	0%	0%	2
**24**	134	100%	0%	0%	2
**36**	123	95.9%	0.8%	3.3%	2
**48**	122	97.5%	0.8%	1.6%	2
**60**	116	96.6%	0.9%	2.6%	2

#: Number

Over 97% of these cells were fully recovered by the segmentation algorithm (Fig 7B). Out of the 340 cells the algorithm could not recover only 9 cells, which had from 65.5 to 85.9% of their seed removed. On average it took 2.6 ± 2.6 (N = 331) time points for the segmentation algorithm to correct segmentation mistakes and the time points required to correct the seed error increased with the severity of the perturbation (Fig 7C). These results demonstrate that our algorithm prevents propagation of segmentation errors by automatically correcting them in subsequent frames, and, thus, is well suited for long-term imaging.

Note that the robustness of the algorithm to perturbations is also exploited in the automatic seeding step. Even if the watershed lines produce seeds that are away from the real cell boundary, our algorithm can use those as seed and converge onto the real cell boundaries (Fig 1A). Also, when a cell is over-segmented, i.e. divided into multiple pieces, each piece acts like a perturbed seed and converge onto the correct segmentation. This is why such pieces overlap significantly after running the segmentation subroutine several times (Figs 1D and 2C).

Next, we tested the robustness of our algorithm with respect to the time interval between successive images. We used cycling budding yeast cells in rich medium (i.e. SCD) imaged with 40X objective. Specifically, we used a correctly segmented image as a seed to segment another image that is taken with a 3-60-minutes time interval and calculated the segmentation accuracy for each case. We scored segmentations that are 90–95% correct as a minor error and we scored segmentations that have a greater error or are lost as a major error. For these test images, the segmentation accuracy is 100% when the images are less than 24 minutes apart, however, it decreases with increasing time interval between the seed and the image to be segmented (Table 4). Note that 60 minutes is a significant time interval for following cycling budding yeast cells, since their doubling time is about 90 minutes in glucose [37]. Thus, we believe that time intervals up to 12 minutes are more efficient for following actively cycling cells.

### Utilizing fluorescent channels that are not dedicated to segmentation to improve image contrast

A common way to improve segmentation accuracy is to mark cell boundaries by fluorescent dyes or markers [17]. However, such techniques occupy fluorescent channels solely for segmentation, increase the risk of phototoxicity, and/or complicate the experimental setup due to added requirements with respect to cloning (fluorescent proteins) or chemical handling (dyes).

It is therefore desirable to limit the number of fluorescent channels dedicated to segmentation.

**Fig 8:**
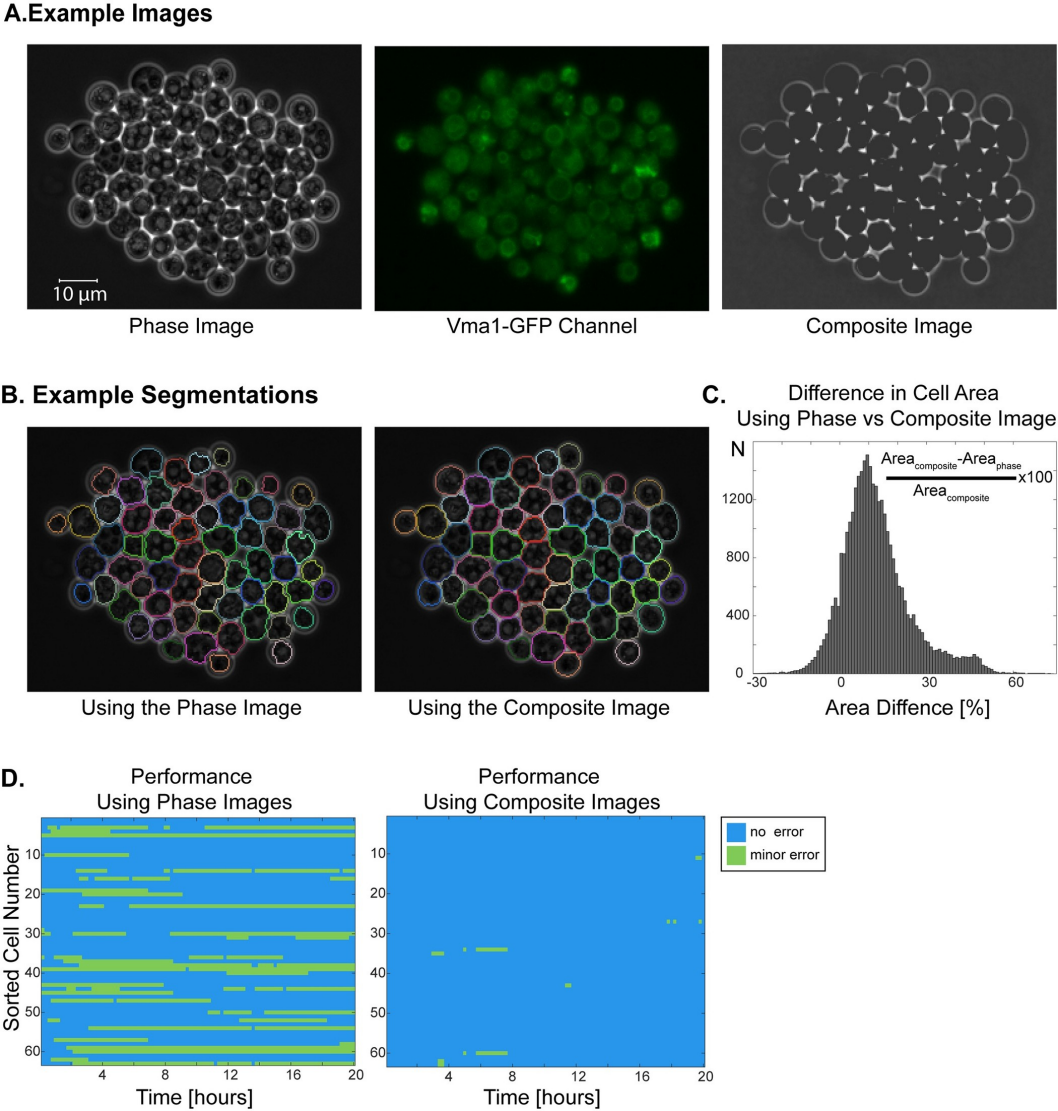
Utilizing a fluorescent channel for improving the segmentation of sporulating cells. **(A)** Example phase image, GFP-channel image and the composite image. In the phase image, spores have very bright patches unlike cycling cells. The composite image is created using the phase and GFP-channel images. Note that Vma1-GFP channel is not dedicated to segmentation. **(B)** Segmentation results using the phase image and using the composite image. Using the composite image corrects for the slight out of focus phase image and significantly improves the segmentation. **(C-D)** Comparison of segmentations with phase and composite images. Example cells were imaged for 20 hours (100 time points) and segmented with phase or the composite images. **(C)** Out of 32868 cell segmentation events, 89.5% of them have a greater area when the composite image is used for segmentation. **(D)** Comparison of errors in segmentation with phase or composite images. Blue no error, green minor error. Minor errors decreased significantly when composite images were used for segmentation.

Nonetheless, *if* any proteins whose localization is at least partially cytoplasmic are fluorescently tagged (dedicated to some biological process of interest), then they can potentially be used to improve the segmentation. Since a large fraction of all proteins exhibit at least partial cytoplasmic localization [38], this is a quite common situation. To take advantage of such cases we developed a method that integrates multi-channel data into the segmentation algorithm. Specifically, this is done by forming a composite image of the phase image (Fig 8A) and the fluorescent channel (Fig 8B), which has high contrast between cell interior and the boundary (Fig 8C).

To test this approach, we applied it to yeast cells imaged through the process of spore formation. Such cells, unlike cycling and mating pheromone treated cells, exhibit regions with high phase contrast (white) within the cells (Fig 8A). Moreover, sporulating cells also exhibit morphological changes when the ellipsoidal yeast alters shape to the characteristic tetrahedral ascus shape [39]. Here we used a strain, where the Subunit A of the V1 peripheral membrane domain of the vacuolar ATPase, *VMA1*, is tagged with GFP marking the vacuole boundaries [40]. Note that this biomarker is not dedicated to segmentation; thus, it is a good trial candidate to explore how our method improves segmentation using a biomarker that is not dedicated to segmentation.

We picked two example fields of view, which are segmented over 20 hours (100 time points), amounting to 32868 segmentation events. We segmented these using phase images or composite images. Next, we scored the errors manually and compared the cell areas for each segmentation event that was correctly segmented by both images. We found that 99.3% of the correctly segmented cells had a different cell area and on average they had 12.8 ± 12.0% bigger cell area when composite images are used (Table 5, Fig 8C, S8 and S9 Movies). More specifically, we found that 89.5% of the cells have a bigger area when composite image is used for segmentation; 0.7% had the same area, and 9.9% had less cell area. The size distributions of cells segmented using phase and composite images were significantly different (two-sample Kolmogorov-Smirnov test, p<0.001).

**Table 5:** Cell area with phase image and composite image.

	% of segmentations with area difference	% cell Area Gain (including negatives and zeros)		Pixel Gain		# Data Points	# Fields of View
		Mean	Std	Mean	Std		
**Sporulating** **Cells**	99.3%	12.8%	12.0%	45.4	39.8	32868	2

**#**: Number

In addition, the accuracy of segmentation improved significantly by using composite images. To quantify the accuracy of segmentation, we scored manually the errors in an example field of view, which was segmented with phase images or composite images. A cell is considered accurately segmented if over 95% of its area was segmented correctly. If a segmentation was 90–95% correct, we labeled it as a minor error. Using composite images, the fraction of correctly segmented cells increased from 75.9% to 99.4% (Table 6, Fig 8D). We found that using the composite image corrects segmentation mistakes that arise due to slightly out of focus phase images.

**Table 6:** Algorithm performance with phase image and the composite image.

Method Used	Phase Only	Composite Image
**# of segmented cells**	63	63
**# of segmentation events**	6300	6300
**Fraction of accurate segmentations**	75.9%	99.4%
**Fraction of minor segmentation errors**	24.1%	0.6%
**Fraction of individual time-series without any errors**	52.4%	87.3%
**# Fields of view**	1	1

**#**: Number

### Bright-field images

Bright-field images are widely used for live-cell imaging, however they are often low contrast and unevenly illuminated [28]. Thus, it is harder to accurately segment cells using bright-field images.

**Fig 9:**
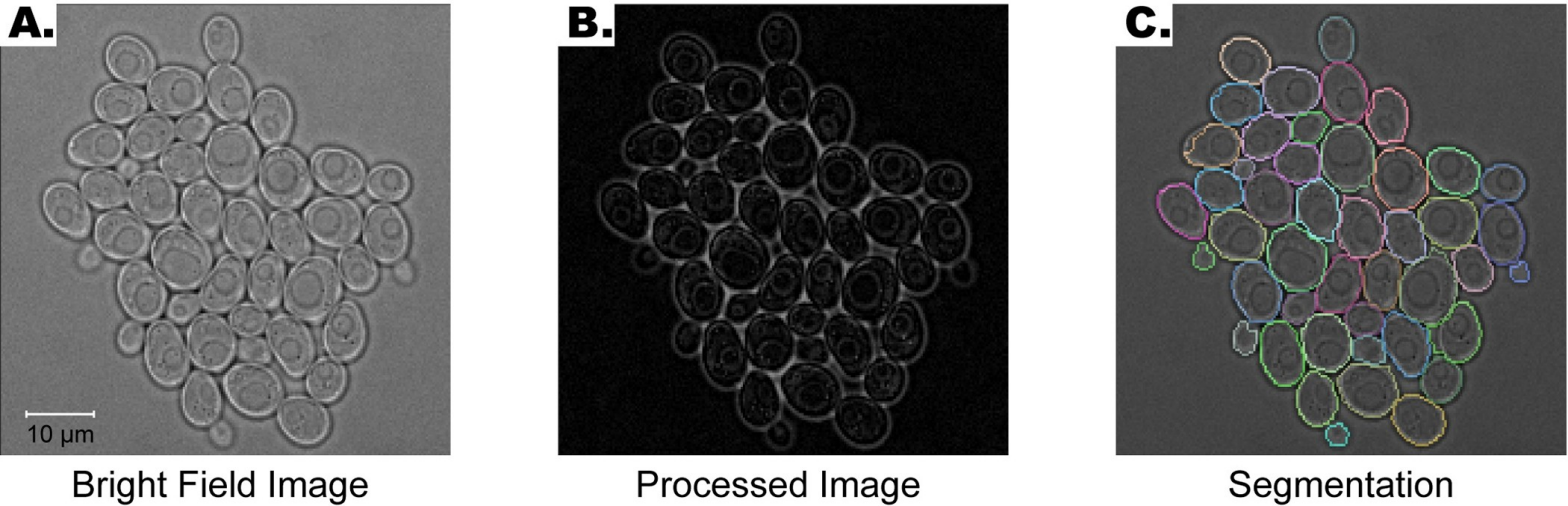
Segmentation of bright-field images. **(A)** Example bright-field image. **(B)** Bright-field image is processed before segmentation by applying a top-hat transform to its complement. **(C)** Segmentation of the image. Each cell boundary is marked with a different color.

To test our algorithm on bright-field images, we segmented two example fields of view imaged with bright-field for five hours (100 time points) (Fig 9A, S10 Movie). First, we processed the bright-field images to make the cell boundaries more prominent. To this end, we applied top-hat transformation to the complement of the bright-field images (Fig 9B) [41]. For details see S1 Text. We were able to successfully segment bright-field images using our segmentation algorithm (Fig 9C; See section *Overall performance* for quantification of errors).

### Overall performance

To rigorously test our segmentation algorithm, we segmented 9 different example cases and evaluated our algorithm’s performance. The errors were scored manually. We counted a cell as ‘correctly segmented’ if over 95% of its area was segmented correctly. If the segmentation was 90–95% correct, we labeled it as a minor error. The rest of the errors, including tracking errors, are called major errors.

**Fig 10:**
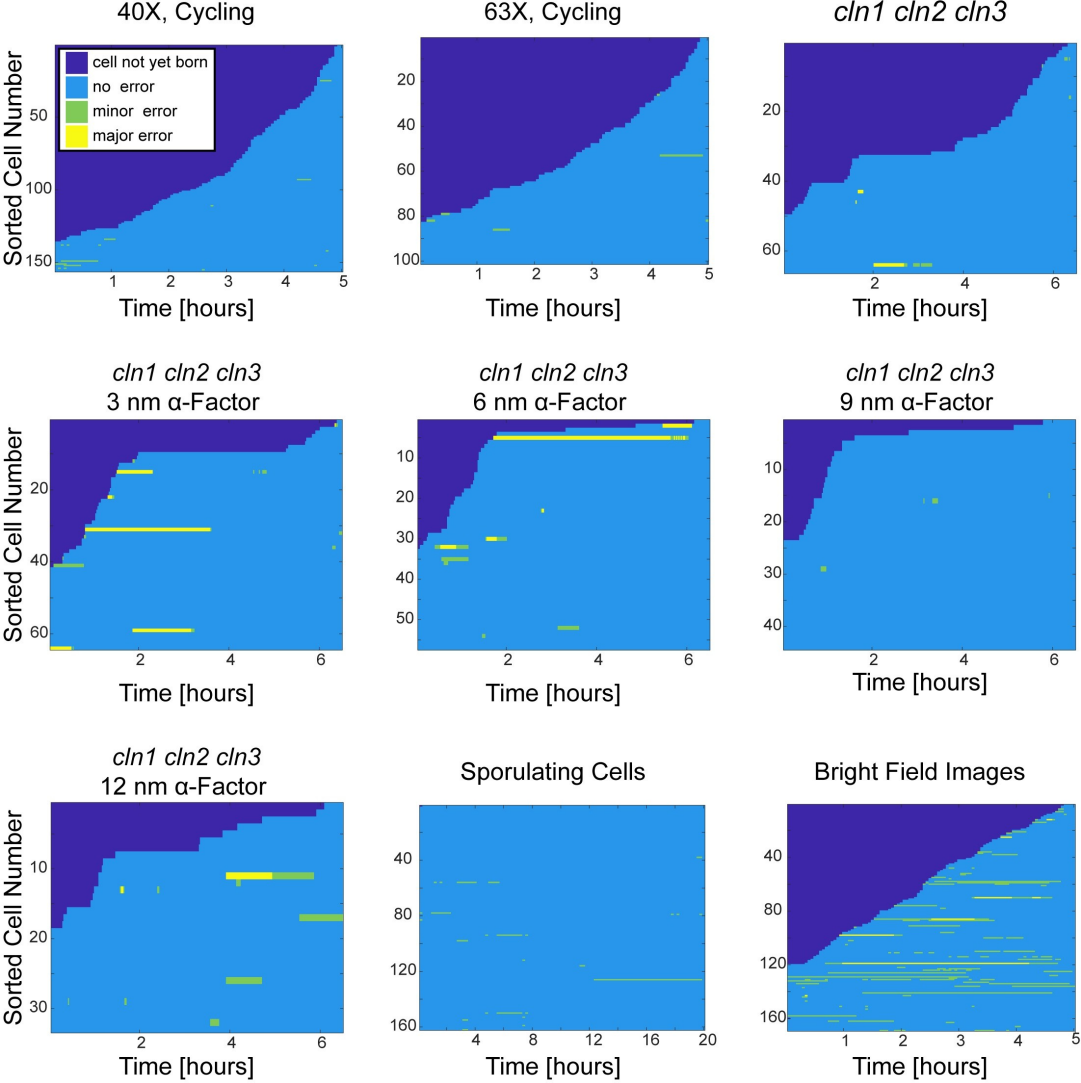
Overall performance of segmentation examples. Sorted cell traces for every example case. Time points where the cell is not yet born are dark blue. Correct segmentations are labeled blue, minor errors green and major segmentation errors yellow. The errors were scored manually. For quantification see Table 7.

The performance of the algorithm is presented in Table 7 and Fig 10. In all example cases at least 92% of the segmentation events were correct. This reached to 99% for some of the example cases. These results demonstrate that our algorithm reaches high accuracy at diverse budding yeast segmentation applications.

**Table 7:** Overall performance of all example cases.

	40X cycling	63X cycling	40X sporulating	0 nM	3 nM	6 nM	9 nM	12 nM	Bright Field
**Total # of segmented cells**	156	101	162	66	64	57	44	33	169
**Total number of segmentation events**	6957	5030	16199	9903	13122	13030	10134	6908	11116
**Fraction of accurate segmentations**	99.4%	99.4%	99.4%	99.4%	97.9%	97.7%	99.9%	97.6%	92.0%
**Fraction of minor segmentation errors**	0.6%	0.6%	0.6%	0.3%	0.4%	0.7%	0.1%	1.8%	6.9%
**Fraction of major segmentation errors**	0%	0%	0%	0.3%	1.7%	1.6%	0%	0.6%	1.1%
**Fraction of individual time-series without any errors**	92.9%	95.0%	90.7%	90.9%	81.25%	84.2%	93.2%	78.8%	63.3%
**# fields of view**	2	3	2	3	2	3	3	3	2

0, 3, 6, 9, 12 nM refer to α-factor concentrations used for treating *cln1cln2cln3* cells. #: Number

Next, to compare our algorithm to other available segmentation algorithms, we tested it on a publicly available benchmark [26] (See also yeast-image-toolkit.biosim.eu). This benchmark provides raw bright field images taken with 100X objective and the ground truth consisting of the location of the cell centers. Based on this ground truth, a segmentation is scored as correct if its center is less than a specified distance away from the ground truth. Briefly, the quality of segmentation and tracking are evaluated using the following measure: Let G be the number of elements in the ground truth, C be the number of elements that are correctly segmented/tracked, and let R be the number of elements in the algorithm. The F-measure is defined as:

$$F=\frac{2C}{R+G}$$

Note that the ratio C/G gives a measure for how much of the ground truth is recovered by the algorithm, however, it does not give information about false positives, i.e. elements in the algorithm result that is not in the ground truth. Likewise, C/R indicates how much of the algorithm output is correct, however, it does not tell us about the false negatives, i.e. elements that are in the ground truth that are not recovered by the algorithm. F-measure is a combined quality measure that takes into account both false positives and false negatives. For further details on the dataset and evaluation criteria see [26].

We applied our algorithm to three datasets available in this benchmark. We omitted datasets with large movements, since our algorithm assumes moderate cell movement between frames. Using F-measure, we show that our segmentation algorithm does as good as the best algorithm reported in [26] on these datasets (Table 8). Note that, as in the section *Bright-field images*, the images are pre-processed for segmentation and tracking (See S1 Text).

**Table 8:** Comparison to other algorithms.

	Segmentation Quality		Tracking Quality		Long-term Tracking Quality^#^	
	Our Algorithm	Best algorithm from [26]*	Our Algorithm	Best algorithm from [26]*	Our Algorithm	Best algorithm from [26]*
**TS1**	0.99	0.99	0.99	0.99	1.00	1.00
**TS2**	1.00	0.99	1.00	0.99	1.00	1.00
**TS6**	0.97	0.96	0.97	0.96	0.98	1.00
**Average**	0.99	0.98	0.98	0.98	0.99	1.00

*based on Table 1 in [26].

^#^For the evaluation of long-term tracking, only cells that are present at all time points are considered.

## Discussion

**Fig 11:**
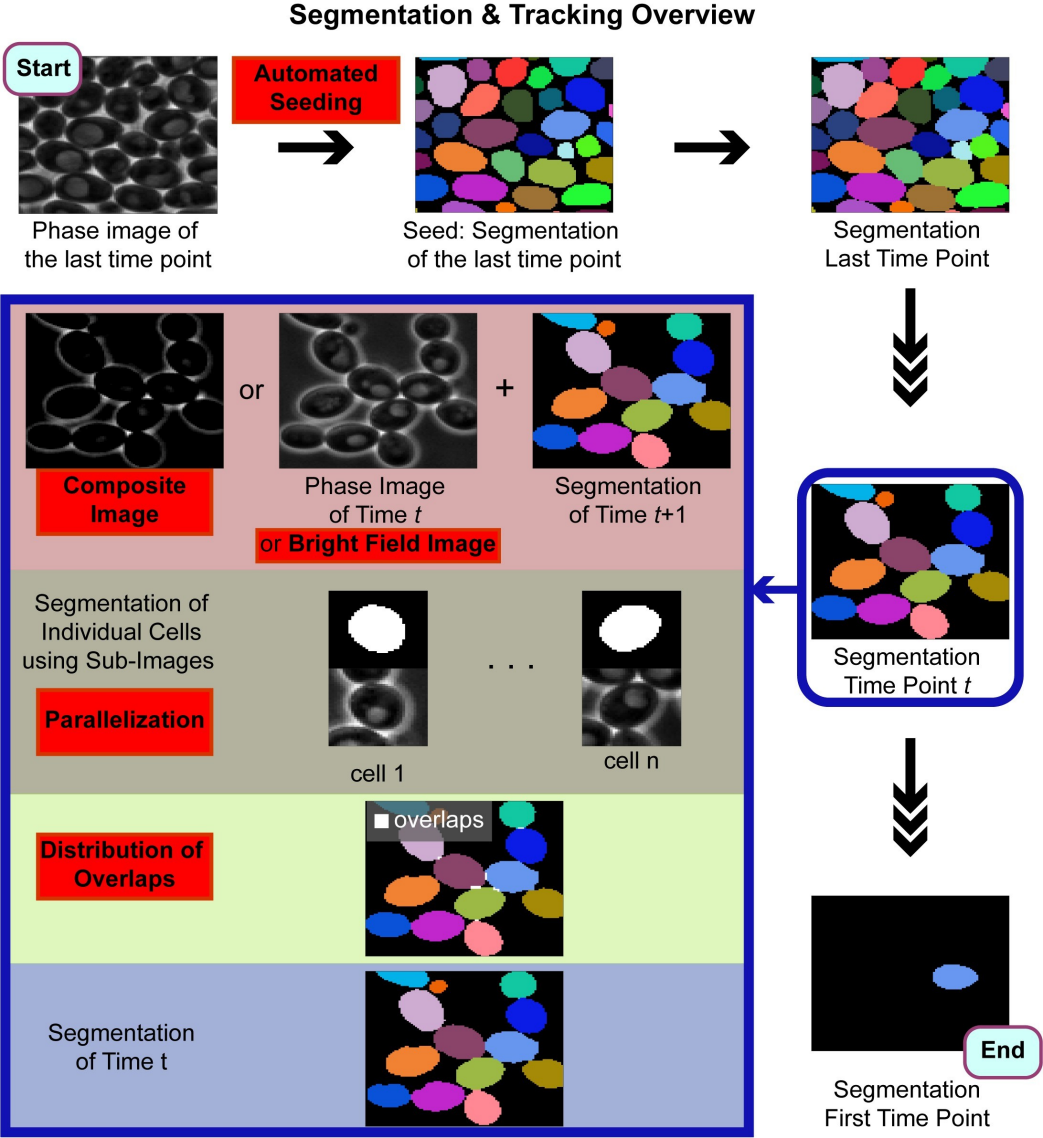
Overview of the segmentation and tracking algorithm. First, the automated seeding step segments the image of the last time point. This seed is fed into the algorithm, which segments the images backwards in time and uses the segmentation of the previous time point as a seed for segmenting the next time point. The segmentation at a given time point is summarized in the blue box. Improvements over the previously published algorithm [30] are highlighted in red boxes.

The generation of single cell data from live-cell imaging relies on accurate segmentation and tracking of cells. Once accurate segmentation is achieved, single-cell data can be extracted from a given image time-series [42]. Here we introduce a fully automated and parallelizable algorithm that accurately segments budding yeast cells with arbitrary morphologies imaged through various conditions (phase / bright field) and objectives (40X/63X/100X). This algorithm improves the accuracy and the speed of our previously published one [30] and adapts it to segmentation of different yeast cell morphologies and imaging conditions (Fig 11, improvements are highlighted in red boxes.). In addition, we developed a novel seeding step, which replaces the semi-automatic seeding of the previous algorithm and enables us to have a fully automatic segmentation algorithm. Since our algorithm can work with no user input, it can be used for large scale single-cell screens.

The automated seeding has two steps: the first one preprocesses the image and prepares it for watershed segmentation. This step provides coarse seeds that are fine-tuned and automatically corrected in the second step of the automated seeding algorithm. This correction of the coarse seed is achieved by utilizing the robustness of our algorithm, i.e. its ability to automatically correct segmentation mistakes at subsequent time points as explored in the section *Robustness of Segmentation*. We exploited this property for automated seeding by running our segmentation subroutine consecutively on the coarse segmentation, where the segmentation result of each step is used as the seed of the next step. This resulted in a novel method that achieves automated cell boundary correction (Fig 2A). In addition, this robustness property enabled us to detect over-segmentation mistakes, since all pieces converge to the correct cell segmentation after application of our segmentation subroutine several times (Fig 2C). Under-segmentation mistakes are detected by generating a subroutine that incorporates the pre-processing step with multiple thresholds. In conclusion, the automated seeding algorithm incorporates novel approaches for cell boundary fine-tuning, and automated under- and over-segmentation detection and automated correction.

The algorithm presented here runs significantly faster than our previous algorithm through parallelization. Even in the absence of a computer cluster, significant time gain can be achieved on a personal computer with two or four processors.

Parallel segmentation of individual cells sometimes leads to assignment of the same pixels to the segmentations of neighboring cells due to false boundaries created by phase halos. Here the algorithm distributes such overlapping initial segmentations, instead of discarding them, which increased the cell area by 1.4–2.8%. The effect of this distribution of initial segmentations is more prominent when cells are densely packed and when the cell size is small (Figs 4 and 5). Although the improvement in cell area translates into a small percentage of area gain, it actually presents a significant improvement in the segmentation of cell boundaries. Thus, the distribution of overlapping initial segmentations increases the accuracy of quantification of fluorescent markers, especially if they are enriched at the cell boundaries. In addition, it enables accurate quantification of biomarker amounts at the cell periphery (Fig 6 and Table 3).

Another aim in budding yeast segmentation is to limit the use of fluorescent markers and dyes. Here we show how fluorescent channels that are devoted to a biological process of interest and not to segmentation, can be exploited to significantly improve the segmentation. The information about the cell location from the fluorescence of the tagged protein and/or autofluorescence of the cells can be incorporated into the phase images by forming composite images using fluorescent channels. In this way, we show a way to utilize existing information about the cell locations in other channels.

To rigorously test our algorithm, we created a comprehensive selection of example cases by including various imaging conditions (phase/bright field), various objective magnifications (40X/63X), and yeast cells with irregular morphologies (sporulating and pheromone arrested cells) (see *Overall Performance*). In addition, we tested our algorithm on a selected subset of a publicly available benchmark [26] (yeast-image-toolkit.biosim.eu). We thank the founders of this benchmark for providing annotated test sets and enabling the community to easily compare algorithms. This benchmark enabled us not only to compare the algorithms, but also to compare the diversity of test sets used and the evaluation criteria applied in testing algorithms. As to diversity, note that although the benchmark successfully incorporates various bright field time series of cycling cells imaged with 100X magnification, it lacks other example cases we covered, including phase images, yeast cells with irregular morphologies, and images with different objective magnifications. As to evaluation criteria, the benchmark criterion accepted a segmentation as correct if its center is less than a specified distance from the manually curated cell center and thus, it does not asses the segmentation accuracy at the cell boundaries. Unlike this criterion, we judged our segmentations at the pixel level, thus we also detected under-segmentation, over-segmentation and local segmentation mistakes that can be missed by the evaluation criterion of the benchmark [26]. Thus, most errors reported as minor in Table 7 would have been counted as correct based on the evaluation criterion of the benchmark. Our strict evaluation manifests itself in the segmentation accuracy of our algorithm on our bright-field test set and the bright-field test sets from the benchmark: note that the fraction of correct segmentations on our own bright-field dataset is 92% (Table 7, last column). However, this fraction is 99% on the benchmark dataset (Table 8).

In our experimental setup the cells are sandwiched in a microfluidics chamber (see *Cell culture and microscopy*) and can only spread out laterally due to budding. This moderate movement enables our algorithm to track the cells based on the overlap between the seed (i.e. segmentation at the previous time step) and the cell location on the next frame. Under such restricted movement conditions, our algorithm is capable of very reliable tracking, as shown by the lack of or very low percentage of major errors, which include tracking errors (Table 7) and by the tracking and long-term tracking quality (Table 8). However, if there is a large movement between the frames, for example due to frame rate being low compared to the growth rate or due to movement of a poorly trapped cell by fluid flow, the segmentation and tracking accuracy goes down. Such cases are beyond the scope of the current manuscript and constitute a future direction.

Overall, given the versatility, speed and accuracy of our algorithm, we believe that it will improve long-term live cell imaging studies in numerous contexts.

## Materials and methods

### Algorithm outline

See S1 Text for algorithm outline and the software.

### Media

SCD (1% succinic acid, 0.6% sodium hydroxide, 0.5% ammonium sulfate, 0.17% YNB (yeast nitrogen base without amino acids/ammonium sulfate), 0.113% dropout stock powder (complete amino acid), 2% glucose, YNA [43] (0.25% yeast extract, 2% potassium acetate)

### Cell culture and microscopy

The images were taken with a Zeiss Observer Z1 microscope equipped with automated hardware focus, motorized stage, temperature control and an AxioCam HRm Rev 3 camera. We used a Zeiss EC Plan-Neofluar 40X 1.3 oil immersion objective or Zeiss EC Plan-Apochromat 63X 1.4 oil immersion objective. The cells were imaged using a Y04C Cellasic microfluidics device (http://www.cellasic.com/) using 0.6 psi flow rate. Cells were kept at 25°C. For details of the strains see Table 9.

**Table 9:** *Saccharomyces cerevisiae* strains.

Name	Genotype	Source
*PK220*	*MAT a/MATα*, *his3/his3*, *trp1/trp1*, *LEU2/leu2*, *ura3/ura3*, *IME1/ime1 pr*::*IME1pr-NLS-mRuby3-URA3*, *WHI5/WHI5-mKOκ-TRP1*, *VMA1/VMA1-mNeptune2*.*5-kanMX*, *ERG6/ERG6-mTFP1-HIS3*	Doncic Lab
*JS264-6c*	*MATa bar1*::*URA3 cln1*::*HIS3 cln2Δ cln3Δ*::*LEU2 ADE2 trp1*::*TRP1- MET3pr-CLN2 FAR1-Venus-kanMX WHI5-mCherry-spHIS5*	[45]
*YL50*	*MAT a/MATα*, *his3/his3*, *trp1/trp1*, *LEU2/leu2*, *ura3/ura3*, *BAR1/bar1*::*Ura3*, *IME1/ime1 pr*::*IME1pr-NLS-mCherry-URA*, *WHI5/WHI5-mKOκ-TRP1*, *VMA1/VMA1-GFP-HIS*, *FAR1/Far1*::*kanMX*	Doncic Lab

*JS264-6c* is isogenic with W303 (*leu2-3*,*112 his3-11*,*15 ura3-1 trp1-1 can1-1*) and *PK220* and *YL50* are with W303 (ho::LYS2 ura3 leu2::hisG trp1::hisG his3::hisG) except at the loci indicated.

#### Cycling cells

*PK220* cells were imaged in SCD every 3 min with 40X or 63X objective, either with phase contrast or bright field. Exposure times are 40 ms for 40X phase and 40X TFP channel, 80 ms for 63X phase, 100 ms for 63X TFP channel and 20 ms for 40X bright field.

#### Sporulating cells

*YL50* cells were imaged in YNA every 12 min. For details of the sporulation protocol see [44]. Exposure times are 15 ms for phase and 30ms for the GFP channel.

#### Pheromone treated cells

*JS264-6c* cells received 1h SCD, then they received SCD for 5.5h with mating pheromone (0,3, 6, 9 or 12 nM) and 10X Methionine. Images were taken with 63X objective every 1.5 min.

## Supporting information

S1 Text Supplementary Text. Tutorial and Algorithm Outline.
(DOCX)

S1 Codes and Example Images (ZIP)

S1 Movie Cycling cells imaged with 40X objective. Cells growing in SCD are imaged every 3 min for 5 hours.
(AVI)

S2 Movie Cycling cells imaged with 63X objective. Cells growing in SCD are imaged every 3 min for 5 hours.
(AVI)

S3 Movie *cln1cln2cln3* cells. Cells growing in SCD are imaged every 1.5 min for 6.5 hours.
(AVI)

S4 Movie *cln1cln2cln3* cells exposed to 3 nm α-factor. The mutant *cln1cln2cln3* cells were grown in SCD for 1 h, and then exposed to 3nM of mating pheromone for 5.5h. The images are taken every 1.5 min.
(AVI)

S5 Movie *cln1cln2cln3* cells exposed to 6 nm α-factor. The mutant *cln1cln2cln3* cells were grown in SCD for 1 h, and then exposed to 6nM of mating pheromone for 5.5h. The images are taken every 1.5 min.
(AVI)

S6 Movie *cln1cln2cln3* cells exposed to 9 nm α-factor. The mutant *cln1cln2cln3* cells were grown in SCD for 1 h, and then exposed to 9nM of mating pheromone for 5.5h. The images are taken every 1.5 min.
(AVI)

S7 Movie *cln1cln2cln3* cells exposed to 12 nm α-factor. The mutant *cln1cln2cln3* cells were grown in SCD for 1 h, and then exposed to 12nM of mating pheromone for 5.5h. The images are taken every 1.5 min.
(AVI)

S8 Movie Sporulating cells. Sporulating cells in YNA are imaged every 12 min for 20 h.
(AVI)

S9 Movie Comparison of using composite images vs phase images. Left is the segmentation of cells using composite images and right are the segmentation of cells using phase images.
(AVI)

S10 Movie Bright Field Images. Cells growing in SCD are imaged every 3 min for 5 hours.
(AVI)

S11 Movie Video tutorial for using the software. (MP4)
