# Supplementary material for: A fully-automated, robust, and versatile algorithm for long-term budding yeast segmentation and tracking
Source: PLoS One. 2019 Mar 27;14(3):e0206395. doi: 10.1371/journal.pone.0206395 (PMC6436761; doi:10.1371/journal.pone.0206395)
Supplement: S1 Text — Tutorial and Algorithm Outline. (DOCX) [file pone.0206395.s001.docx]

**Tutorial**

Here we explain how to use the accompanying segmentation and tracking code aimed at someone with basic knowledge of MATLAB. No image analysis background is required to run the provided code. The algorithm and parameters are discussed later in the *Algorithm outline* section. S11 Movie also demonstrates how to use the provided software.

**File organization**

The supplementary data contains the following:

Scripts

- Automated_Seeding.m
- Segmentation_and_Tracking.m

Functions

- pre_processing_and_watershed.m
- check_divide.m
- retain_largest_object.m
- segmentation_subroutine_for_seeding.m
- segmentation_subroutine.m

Images

- Example_Images: folder containing 20 example TIF images

The main script for automated seeding is Automated_Seeding.m. The seed generated by this code is used by the main script for segmentation and tracking, Segmentation_and_Tracking.m. The code is implemented in MATLAB R2017a.

To run the code without error, scripts, functions and the folder containing the images must be in the same folder. The accompanying code runs on two sets of example images each consisting of 10 images of size 250x250 pixels. We limited the number and the size of the example images to keep the supplementary file size small.

The first set of example images are taken with a 40X objective (img40X_01 to img40X_10) and the second set with a 63X objective (img63X_01 to img63X_10). The same code segments and tracks both example images with a minimal change of parameters, which are indicated below.

**Running the scripts to segment and track the cells**

First open in MATLAB the folder containing the scripts, functions and the image folder.

The default setting for both codes is to run in parallel with 40X example images. If one wants to run them sequentially and/or switch to the 63X example images, the lines to comment out and uncomment are indicated below.

First, run Automated_Seeding.m to generate the seed.

Automated_Seeding.m

This code takes the last image of an image series and segments it. It then saves the result, which will be used as the seed during segmentation and tracking.

To run this code sequentially, comment out the lines at which a parallel pool is opened (line 22) and closed (line 362). Next, comment out parfor (line 77) and uncomment for (line 76).

To segment the example 63X images, comment out the lines 33-38, which specify the image name and parameters for 40X examples. Uncomment the lines 41-46.

After this code is run, the seed for 40X example images will be saved as ‘Example_Seed_40X’ and the seed for 63X will be saved as ‘Example_Seed_63X’. In addition, the result will be visualized in a figure, where the cell boundaries are superimposed in yellow on the phase image.

Next, to segment and track the cells, run Segmentation_and_Tracking.m.

Segmentation_and_Tracking.m

This code takes in the seed generated by Automated_Seeding.m and the phase images to segment and track the cells through time.

To run this code sequentially, comment out the lines at which a parallel pool is opened (line 20) and closed (line 210). Then comment out parfor (line 91) and uncomment for (line 90).

To segment and track the 63X examples, comment out lines 35-41 and uncomment the lines 44-50.

This step generates as the output the structure all_obj, which has the following two fields (also explained in the supplementary material of [1]):

all_obj.cells: This field has dimensions number of rows of the image x number of columns of the image x number of time points. Thus, for the example provided here it has dimensions 250 x 250 x 10. It holds the segmentation results for all time points and cells.

all_obj.twoD_area: This field has dimensions number of cells x number of time points. It holds the cell sizes. The rows correspond to cell numbers and the columns correspond to time points.

**Algorithm outline**

The detailed outline of the segmentation subroutine is given in [1]. Thus, here we will briefly summarize the segmentation subroutine that is introduced in [1] and give the outline of the automatic seeding step and the new steps added to the algorithm.

**Review of the previously published segmentation subroutine**

Since our method builds on our previously published algorithm [1], here we briefly review the key segmentation subroutine the new algorithm uses. This segmentation subroutine focuses on segmenting one cell given a seed for the cell, which is a black and white image containing the approximate location of the cell, and a subimage containing the cell. The subimage is generated by cutting a neighborhood around the seed-location.

To segment the cell, the algorithm first creates a binary image by thresholding the subimage, then applies a distance transform and a watershed transform to the binary image [2-4]. At the thresholding step, instead of choosing one optimal threshold, the algorithm goes through every possible integer threshold for an 8-bit image, i.e. between 1-255, and each threshold gives rise to the segmentation of the subimage into different regions. Any region that overlaps significantly with the seed is added to a composite image with a certain weight. Next, drastic movement between frames and bright phase pixels associated with phase halo are penalized. Finally, pixels that are below a threshold are removed from the composite image, giving the final composite image, which we call ‘*cell score*’. This subroutine is implemented in segmentation_subroutine.m.

We use this segmentation subroutine in the new algorithm as well. In addition, the automatic seeding step relies on a customized version of this subroutine to detect and correct seeding mistakes automatically. See segmentation_subroutine_for_seeding.m.

**Automated seeding**

The automated seeding step has two main parts (Figure 1): (1) pre-processing the image using morphological image analysis and applying watershed transform to the processed image, and (2) fine-tuning the cell boundaries and automatically correcting under- and over-segmentation.

**Pre-processing and watershed**

1. *Image Padding to Improve the Segmentation on Image Edges*

Before processing the phase image, the image is symmetrically padded, i.e. mirror reflections of the image are added to it at all four directions. This has two main advantages: (1) Although generally we are not interested in the cells that are at the boundaries of the field of view, their correct segmentation improves the segmentation of the contiguous cells. (2) We will apply spatial filters [5] such as average filtering and standard deviation filtering to the image in a later step and this padding sets the boundary conditions for these filters.

1. *Finding Cell Colonies: Coarse Foreground – Background Segmentation*

At this step of pre-processing, we find the location of each colony on the phase image, that is, coarsely separate background from the foreground (i.e. cell colonies). First, to reduce the variation in the phase image, we apply opening by reconstruction to the image [5], where the *marker* is an opened version of the phase image and the *mask* is the phase image. Then we binarize the resulting image using the Otsu’s method with adaptive thresholding [6, 7]. After removing small connected components from the binary image, to close the gaps we apply closing by reconstruction [5, 8], where the *mask* is the closed version of the binary image and the *marker* is the binary image. Note that this last step can be done simply with closing too, however, morphological reconstruction gives finer tuned colony boundaries.

1. *Finding the Cell Contours and Interstices: Determining Locally Bright Pixels*

Next, we determine the cell contour pixels and the pixels between cells. Pixels at the colony boundaries are less bright than the ones between cells, thus, to facilitate their detection we increase their intensity by a constant number set by the parameter colony_boundary_bias. Note that colony boundaries are detected using the coarse foreground-background segmentation. Next, we apply average filtering and standard deviation filtering to the image created by opening by reconstruction as in the previous step and determine the pixels that are greater than one standard deviation from the average. Next, we apply closing to the resulting binary image and also remove small connected components to avoid over-segmentation.

1. *Distance Transform and Processing*

The Euclidean distance transform is applied to the binary image holding the cell contours and interstices. Next, we apply median filtering to the transformed image. After the distance transform, the cell interiors have the highest values, since they are furthest away from the cell borders. To convert them from local maxima to local minima, we multiply the transform with negative one. To further process this image, we first find the local minima of this image as a binary image. We dilate the binary image having the local minima so that local minima that are too close to each other merge. Then we remove the minima at colony boundaries. Next, we modify the distance transform so that the remaining minima are the only local minima in the image.

1. *Watershed Transform*

Next, the watershed transform is applied on the processed distance transform from the previous step. We used the watershed algorithm of MATLAB. But note that there are various implementations of the watershed algorithm, and each might give different results, especially at the image plateaus [2, 6, 9]. Tsygankov et al. used their implementation for the segmentation of yeast cells [10].

The pre-processing and watershed step is implemented in the pre_processing_and_watershed.m function.

**Automated fine-tuning and correction**

After the initial segmentation of the phase image, the algorithm automatically fine-tunes the cell boundaries and corrects under- and over-segmentation. A flowchart of this step is given in Figure 1D and example cases are demonstrated in Figure 2.

1. *Refining Boundaries and Checking for Under-segmentation*

For each cell, using the initial segmentation, a region around the putative cell is cut. First, the algorithm checks whether this region has more than one cell, i.e. whether the putative cell should be divided. To check this, we apply a simpler version of the previous pre-processing and watershed step, but with multiple (total of 21) thresholds for finding the cell contour pixels and interstices. This is implemented in the check_divide.m function. If at least 17 of these thresholds agree that a pixel belongs to a cell, then we designate that pixel a cell pixel; otherwise we designate it as a non-cell pixel.

If the putative cell is not divided, then we apply a version of the segmentation subroutine, where the cell is segmented consecutively (Figure 2A), by using the result of the previous segmentation as the seed of the next segmentation. This is implemented in the segmentation_subroutine_for_seeding.m function.

If the putative cell is divided, then the algorithm moves to the blue box in Figure 1D: The putative cell is divided, and segmentation subroutine is applied to each piece. Next, these pieces are checked for overlaps. If the overlap among the pieces is greater than one fourth of the smaller piece area, then the pieces are merged back. If the overlap is less than this threshold, the overlapping area is distributed among the pieces using the scores generated by the segmentation subroutine and the pieces are treated as different cells for the rest of the algorithm.

1. *Checking Overlaps Between the Cell Segmentations*

The previous step generates a score for each cell. In this step overlaps between the cell segmentations are checked: if the overlaps are less than one fourth of the smaller piece area, they are distributed based on the cell scores (Figure 2C); otherwise, two cells are merged (Figure 2D).

**New steps in the segmentation and tracking algorithm**

**Distribution of overlapping initial segmentations**

Segmentation subroutine generates cell scores for each cell. The overlapping regions between the segmentations are distributed among them based on these scores.

This section is implemented in lines 135-159 in Segmentation_and_Tracking.m.

**Removal of the cell areas that are on the background**

The images are coarsely segmented into foreground and background as in the pre-processing step of the automated seeding. Using this, parts of the cell segmentations that are on the background are removed.

This section is implemented in lines 165-194 in Segmentation_and_Tracking.m.

**Formation of the composite image**

This part is not included in the example code; however, its implementation is shown below.

To increase the contrast between cell and non-cell pixels, we use the fluorescent channel image, which is *not* devoted to segmentation, to form a composite image with the phase image as shown in Figure 8A. To this end, we first remove overly bright pixels from the fluorescent channel to avoid noise. Next, we smooth the fluorescent channel image with a Gaussian filter and use this to form the composite image. We implemented this part in the following way:

%First read the images. Next:

IP=double(IP); %phase image

IF=double(IF); %fluorescent channel image

%remove too bright outliers from the fluorescent channel image.

Y=prctile(IT(:),99.9);

IF(IF>Y)=Y;

%Normalize 0-255

IP=((IP-min(IP(:)))./(max(IP(:))-min(IP(:)))).*255;

IF=((IF-min(IF(:)))./(max(IF(:))-min(IF(:)))).*255;

%Gaussian Filtering

IF2=imgaussfilt(IF,1);

IP=uint8(IP); IF=uint8(IF2);

Icomp=(255-IP)+IF;

Icomp=255-((255./max(Icomp (:)-min(Icomp (:)))).*(Icomp-min(Icomp (:)))); %composite image

Note that for the background determination explained in the section *Removal of the cell areas that are on the background* IP is used*.*

**Quantification of the Erg6-TFP at the Cell Periphery**

To process the Erg6-TFP channel images, we first remove extremely bright pixels as shown in the section *Formation of the composite image*. Next, to remove noise we apply contraharmonic mean filter of order 2 to the image using a 3-by-3 square structuring element. Then, we subtract the 98^th^ percentile pixel intensity of the non-cell region, i.e. background, from the whole image to remove the background noise.

We considered pixels that are within 2 units ‘cityblock’ distance to the cell boundary as the cell periphery. It is calculated as follows:

%Given the segmentation of the cell, cell_segmentation, the cell periphery is determined as follows:

dist_to_cell_boundary=bwdist(~(cell_segmentation),'cityblock');

cell_periphery=( dist_to_cell_boundary ==1 | dist_to_cell_boundary ==2);

**Bright-field processing**

Bright field images are briefly processed before being fed to the algorithm.

Implementation of the preprocessing for seeding:

%After reading the bright-field image, IB:

IB=double(IB);

IB_filt=imgaussfilt(IB,6);

IB=IB-IB_filt;

IB=imcomplement(IB);

%Normalize 0-255

IB=((IB-min(IB(:)))./(max(IB(:))-min(IB(:)))).*255;

For the segmentation and tracking algorithm top-hat transformation is applied to the complement of the bright field image (Figure 9), which is implemented in the following way:

%After reading the bright-field image, IB:

IB=double(IB);

IB=imcomplement(IB);

IB=((IB-min(IB(:)))./(max(IB(:))-min(IB(:)))).*255;

IB_unprocessed=IB; %Keep this image for background calculation.

se=strel('disk',4);

I=imtophat(IB,se); %processed bright-field image as shown in Figure 9B.

Note that for the background calculation explained in the section *Removal of the cell areas that are on the background* IB_unprocessed is used*.*

**Description of parameters**

To adapt the segmentation and tracking algorithm to different applications, the parameters need to be adjusted based on the cell sizes and objective magnification. The description of parameters for automated seeding step are given in Table S1. The parameters used in the segmentation subroutine, i.e. max_size_vs_largest_cell, max_area_incrase_per_tp, higher_threshold, lower_threshold, threshold_increase_factor, phase_substraction_factor, min_cell_size and cell_margin are explained in detail in the supplementary material of [1]. The version of this segmentation subroutine adapted for automated seeding has one more parameter, nrep, which sets the number of consecutive segmentations.

**Table S1: Description of parameters used in automated seeding step.**

| **Parameter Name** | **Typical value** | **Explanation** |
| --- | --- | --- |
| size_padding | 80 | Specifies the size of padding.  Before the pre-processing step, the images are padded to improve the segmentation of the cells at the image edges. |
| size_strel_bg_1 | 6 | Specifies the size of the structuring element used for connecting the possible gaps on the cell boundaries during the coarse foreground-background segmentation step. |
| size_strel_bg_2 | 20(40X),  80(63X) | Size of the square structuring element used at the closing step during the coarse foreground-background segmentation. |
| min_colony | 1000 (40X),  1500(63X) | The minimum number of pixels to be considered as a colony of cells during the coarse foreground-background segmentation. |
| colony_boundary  _bias | 40 | Colony_boundary_bias is added to the pixels that are at the colony boundaries during the determination of locally bright pixels. Colony boundaries are determined using the coarse foreground-background segmentation. |
| size_strel_filters | 35 | Size of the square structuring element used for average and standard deviation filters during the determination of locally bright pixels.  This structuring element’s size should be comparable to the object size. |
| cell_boundary_threshold | 1 | Specifies the threshold for determining locally bright pixels, which are selected based on the average and standard deviation of pixel intensities in a neighborhood around each pixel:  Threshold= average + (cell_boundary_threshold)*(standard deviation).  Pixels with a greater intensity than this threshold are labeled as the cell boundary pixels. |
| clean_BW | 10 (40X),  80(63X) | After detecting the cell boundary pixels, connected components of less than clean_BW are removed. This step prevents over-segmentation. |

**Processing and parameters for the benchmark**

The benchmark images are bright-field images taken with 100X objective [11] (yeast-image-toolkit.biosim.eu). The images were processed similar to the section *Bright-field images.*

Implementation of pre-processing for seeding:

%After reading the bright-field image, IB:

IB=double(IP);

IB_filt=imgaussfilt(IB,10); %Since these are 100X images, the smoothing kernel standard deviation is increased.

IB=IB-IB_filt;

%Normalize 0-255

IB=((IB-min(IB(:)))./(max(IB(:))-min(IB(:)))).*255;

After the seeding step, in case there were some faulty seeds, they were corrected or removed.

Implementation of pre-processing for segmentation and tracking:

%After reading the bright-field image, IB:

IB=double(IB);

%normalization and processing of bright-field image for background calculation

IB_bg=IB; %Prepare this image for background calculation.

IB_bg_filt=imgaussfilt(IB_bg,20);

IB_bg = IB_bg - IB_bg_filt;

IB_bg =(( IB_bg -min(IB_bg(:)))./(max(IB_bg(:))-min(IB_bg (:)))).*255; %Normalize 0-255.

%Use IB_bg for background calculation as explained in the section *Removal of the cell areas that are on the background*.

%normalization and processing of bright-field image for segmentation and tracking

IB=((IB-min(IB(:)))./(max(IB(:))-min(IB(:)))).*255;

se = strel('disk',10); % Since these are 100X images, the smoothing kernel standard deviation is increased.

I=imtophat(IB,se); %processed bright-field image

The parameters used for the benchmark images area given in Table S2. Unless otherwise stated, the parameters are the same for the Automated_Seeding and Segmentation_and_Tracking scripts.

**Table S2: Parameters used for benchmark images**

| **Parameter Name** | **Value** |
| --- | --- |
| size_padding | 80 |
| size_strel_bg_1 | 8 (Seeding)  12 (Segmentation and Tracking) |
| size_strel_bg_2 | 70 (Seeding)  100 (Segmentation and Tracking) |
| min_colony | 2000 (Seeding)  1000 (Segmentation and Tracking) |
| colony_boundary_bias | 40 |
| size_strel_filters | 35 |
| cell_boundary_threshold | 1 |
| clean_BW | 30 |
| max_size_vs_largest_cell | 1.5 |
| max_area_increase_per_tp | 0.18 (Seeding)  0.5 (Segmentation and Tracking) |
| higher_threshold | 0.2 |
| lower_threshold | 0.2 |
| threshold_increase_factor | 0.05 |
| phase_subtraction_factor | 1(Seeding)  2(Segmentation and Tracking) |
| dist_modifier | 3 (Seeding)  0.1 (Segmentation and Tracking) |
| min_cell_size | 50 |
| max_cell_size | 2000 |
| cell_margin | 10 |
| nrep | 5 |

**References**

1. Doncic A, Eser U, Atay O, Skotheim JM. An algorithm to automate yeast segmentation and tracking. PLoS One. 2013;8(3):e57970. doi: 10.1371/journal.pone.0057970. PubMed PMID: 23520484; PubMed Central PMCID: PMCPMC3592893.

2. Roerdink JB, Meijster A. The watershed transform: Definitions, algorithms and parallelization strategies. Fundamenta informaticae. 2000;41(1, 2):187-228.

3. Meyer F. Topographic distance and watershed lines. Signal processing. 1994;38(1):113-25.

4. Meyer F. The watershed concept and its use in segmentation: a brief history. arXiv preprint arXiv:12020216. 2012.

5. Gonzalez RC, Woods E. R., 2002. Digital Image Processing. Addison-Wesley.

6. Otsu N. A threshold selection method from gray-level histograms. IEEE transactions on systems, man, and cybernetics. 1979;9(1):62-6.

7. Bradley D, Roth G. Adaptive thresholding using the integral image. Journal of graphics tools. 2007;12(2):13-21.

8. Vincent L. Morphological grayscale reconstruction in image analysis: applications and efficient algorithms. IEEE transactions on image processing. 1993;2(2):176-201.

9. Nikodem J. Plateau problem in the watershed transform. Computing and Informatics. 2012;28(2):195–207.

10. Tsygankov D, Chu P-H, Chen H, Elston TC, Hahn K. User-friendly tools for quantifying the dynamics of cellular morphology and intracellular protein clusters. Methods in cell biology. 2014;123:409.

11. Versari C, Stoma S, Batmanov K, Llamosi A, Mroz F, Kaczmarek A, et al. Long-term tracking of budding yeast cells in brightfield microscopy: CellStar and the Evaluation Platform. Journal of The Royal Society Interface. 2017;14(127):20160705.
