# Supplementary figures and images for: A fully-automated, robust, and versatile algorithm for long-term budding yeast segmentation and tracking
Source: PLoS One. 2019 Mar 27;14(3):e0206395. doi: 10.1371/journal.pone.0206395 (PMC6436761; doi:10.1371/journal.pone.0206395)

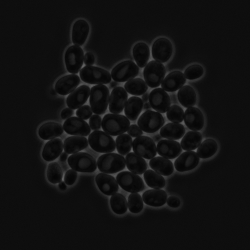

Supplement: S1 Codes and Example Images — (ZIP) [file pone.0206395.s002.zip › Codes_example_images/Example_Images/img40X_01.tif]

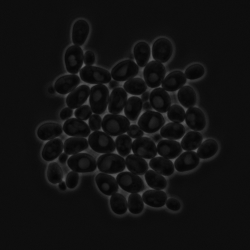

Supplement: S1 Codes and Example Images — (ZIP) [file pone.0206395.s002.zip › Codes_example_images/Example_Images/img40X_02.tif]

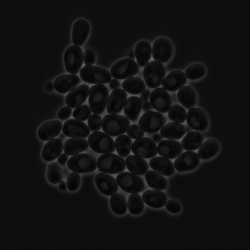

Supplement: S1 Codes and Example Images — (ZIP) [file pone.0206395.s002.zip › Codes_example_images/Example_Images/img40X_03.tif]

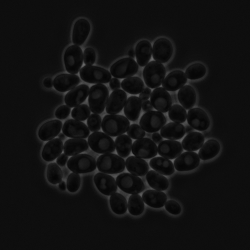

Supplement: S1 Codes and Example Images — (ZIP) [file pone.0206395.s002.zip › Codes_example_images/Example_Images/img40X_04.tif]

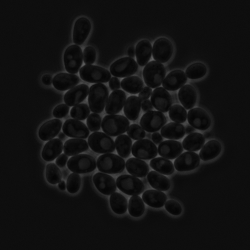

Supplement: S1 Codes and Example Images — (ZIP) [file pone.0206395.s002.zip › Codes_example_images/Example_Images/img40X_05.tif]

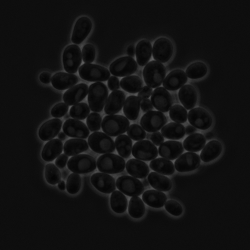

Supplement: S1 Codes and Example Images — (ZIP) [file pone.0206395.s002.zip › Codes_example_images/Example_Images/img40X_06.tif]

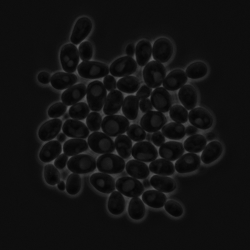

Supplement: S1 Codes and Example Images — (ZIP) [file pone.0206395.s002.zip › Codes_example_images/Example_Images/img40X_07.tif]

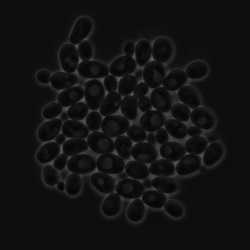

Supplement: S1 Codes and Example Images — (ZIP) [file pone.0206395.s002.zip › Codes_example_images/Example_Images/img40X_08.tif]

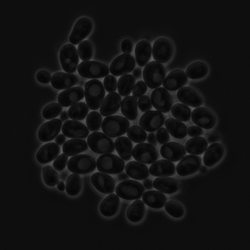

Supplement: S1 Codes and Example Images — (ZIP) [file pone.0206395.s002.zip › Codes_example_images/Example_Images/img40X_09.tif]

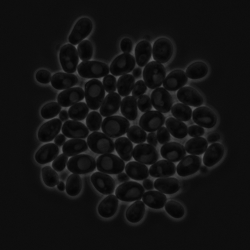

Supplement: S1 Codes and Example Images — (ZIP) [file pone.0206395.s002.zip › Codes_example_images/Example_Images/img40X_10.tif]

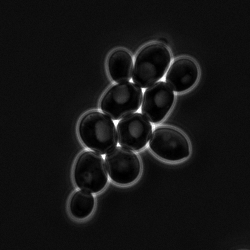

Supplement: S1 Codes and Example Images — (ZIP) [file pone.0206395.s002.zip › Codes_example_images/Example_Images/img63X_01.tif]

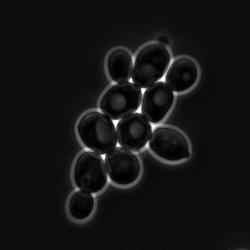

Supplement: S1 Codes and Example Images — (ZIP) [file pone.0206395.s002.zip › Codes_example_images/Example_Images/img63X_02.tif]

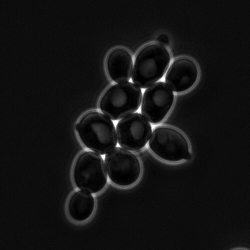

Supplement: S1 Codes and Example Images — (ZIP) [file pone.0206395.s002.zip › Codes_example_images/Example_Images/img63X_03.tif]

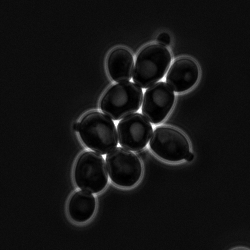

Supplement: S1 Codes and Example Images — (ZIP) [file pone.0206395.s002.zip › Codes_example_images/Example_Images/img63X_04.tif]

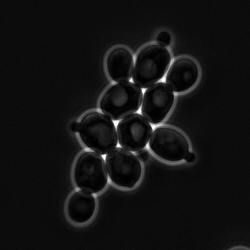

Supplement: S1 Codes and Example Images — (ZIP) [file pone.0206395.s002.zip › Codes_example_images/Example_Images/img63X_05.tif]

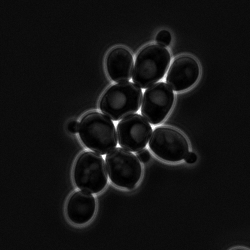

Supplement: S1 Codes and Example Images — (ZIP) [file pone.0206395.s002.zip › Codes_example_images/Example_Images/img63X_06.tif]

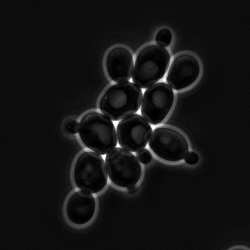

Supplement: S1 Codes and Example Images — (ZIP) [file pone.0206395.s002.zip › Codes_example_images/Example_Images/img63X_07.tif]

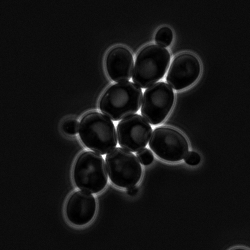

Supplement: S1 Codes and Example Images — (ZIP) [file pone.0206395.s002.zip › Codes_example_images/Example_Images/img63X_08.tif]

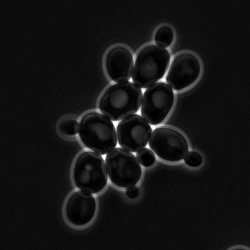

Supplement: S1 Codes and Example Images — (ZIP) [file pone.0206395.s002.zip › Codes_example_images/Example_Images/img63X_09.tif]

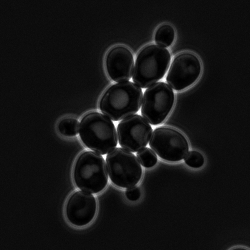

Supplement: S1 Codes and Example Images — (ZIP) [file pone.0206395.s002.zip › Codes_example_images/Example_Images/img63X_10.tif]
